# Comprehensive genomic exploration of class III peroxidase genes in guava unravels physiology, evolution, and postharvest storage responses

**DOI:** 10.1038/s41598-024-51961-4

**Published:** 2024-01-16

**Authors:** Shaista Gull, Muhammad Moaaz Ali, Shaghef Ejaz, Sajid Ali, Majeeda Rasheed, Ahmed Fathy Yousef, Piotr Stępień, Faxing Chen

**Affiliations:** 1 Department of Horticulture, Bahauddin Zakariya University, MultanPunjab, 66000 Pakistan; 2 College of Horticulture, Fujian Agriculture and Forestry University, Fuzhou, 350002 China; 3 Khwaja Fareed University of Engineering and Information Technology, Rahim Yar Khan, Pakistan; 4 Department of Horticulture, College of Agriculture, University of Al-Azhar (Branch Assiut), Assiut, 71524 Egypt; 5 Institute of Soil Science, Plant Nutrition and Environmental Protection, Wrocław University of Environmental and Life Sciences, Ul. Grunwaldzka 53, 50-357 Wrocław, Poland

**Keywords:** Plant molecular biology, Plant physiology, Secondary metabolism

## Abstract

Peroxidases (PRXs) play multifaceted roles in plant growth, development, and stress responses. Here, we present a comprehensive analysis of the *PRX* gene family in guava, a globally significant fruit. In the guava genome, we identified 37 *PRX* genes, a number lower than that of Arabidopsis, suggesting a distinctive gene family expansion pattern. Phylogenetic analysis unveiled close relationships with Arabidopsis *PRXs*, with 12 *PgPRX* genes forming ortholog pairs, indicating a specific expansion pattern. Predictions placed most PRX proteins in the chloroplast and extracellular regions. Structural analysis of PgPRX proteins revealed commonalities in domain structures and motif organization. Synteny analysis underscored the dynamic role of segmental duplication in the evolution of guava's *PRX* genes. We explored the dynamic expression of *PgPRX* genes across guava tissues, exposing functional diversity. Furthermore, we examined changes in peroxidase levels and gene expressions during postharvest fruit storage, providing insights for preserving fruit quality. This study offers an initial genome-wide identification and characterization of Class III peroxidases in guava, laying the foundation for future functional analyses.

## Introduction

Peroxidases (PRXs) are a group of isozymes that play a crucial role in regulating the growth and life cycles of plants, with a particular emphasis on their contribution to plant defense processes^1^. These PRXs can be categorized based on their protein structures into two main subgroups: non-hemoglobin PRXs and hemoglobin PRXs^2^. Hemoglobin PRXs can be further classified into three distinct classes based on their catalytic properties and sequence characteristics: Class I, Class II, and Class III peroxidases. Class I peroxidases are widely distributed among various organisms, excluding animals. They serve a critical role in safeguarding cells by removing excessive H_2_O_2_, thereby preventing cell damage^3,4^. Class II peroxidases are predominantly associated with the decomposition of lignin and are primarily prevalent in fungi^5^. On the other hand, Class III peroxidases (PRX, EC 1.11.1.7) constitute a multigene family found in various plant species, as recognized by multiple researchers^6–8^. PRX proteins exhibit a remarkable conservation of amino acid residues, including the presence of the protoporphyrin IX domain^9^. Additionally, many plant PRX proteins undergo glycosylation, wherein they bind to carbohydrate side chains. This glycosylation process serves to protect the proteins from degradation by proteases, thereby ensuring the stability of these enzymes^10^. The distal histidine plays a crucial role in the catalytic activities of PRX proteins. It is formed through the interaction of two histidine residues with a heme group and eight cysteine residues^11^.

Recent research has highlighted the significance of PRX proteins in various physiological processes. This includes their involvement in the regulation of growth hormone metabolism, cell wall integrity, cell elongation, lignin formation, lipid cell development, and their contribution to enhancing tolerance to both biotic and abiotic stresses^12–17^. In Arabidopsis, peroxidase genes *AtPrx33* and *AtPrx34* have been associated with root elongation^18^. Another gene, *AtPrx72*, was found to play a role in regulating lignification in plant cells^19^. In cotton, the GhPOX1 protein is known to regulate reactive oxygen species, contributing to the elongation of cotton fibers^20^. An analysis using microarrays in maize revealed a direct association between the relative expression levels of *PRX* genes and their responses to abiotic stress. This observation suggests that *PRXs* play a pivotal role in mitigating the effects of abiotic stress^21^. Furthermore, it is noteworthy that the activity of specific isoforms of PRX proteins shows a significant correlation with specific cellular processes and their distinct localization within the cell^22,23^.

Genome-wide studies serve as valuable tools for comprehending the attributes of extensive multi-genic families. Recently, investigations have explored the *PRX* gene family and its involvement in various physiological processes across several species, encompassing Arabidopsis^6^, rice^11^, maize^21^, potato^8^, Chinese pear^24^, sweet oranges^25^, and passion fruit^26^. However, thorough characterization of *PRXs* is not yet studied in guava. Guava (*Psidium guajava* L.) is a nutritious fruit, mainly grown in Pakistan, Brazil, South Africa, Mexico, India, Venezuela, Egypt and Columbia^27^. In nature, guava is climacteric; although its few cultivars are non-climacteric^28,29^. Under cold storage, at 85–90% relative humidity and 8–10 °C temperature, it is possible to store guava fruits up to 30 days^30^.

The availability of guava (*Psidium guajava*) genome published by Feng et al.^31^ facilitated genomic, proteomic, and functional studies. To enhance our understanding of the *PRX* gene family in guava, we systematically identified a total of 37 *PRXs*. Subsequently, we delved into their phylogenetic relationships, subcellular localization, and gene duplication events. Additionally, we explored the expression patterns of these *PRX* genes in various guava tissues, including the root, stem, leaf, and flower. Furthermore, we conducted a detailed examination of the expressions of *PgPRXs* in postharvest stored guava fruits under two distinct storage conditions: ambient storage (at 25 ± 2 °C, 40–60% RH) and cold storage (at 4 ± 1 °C, 85 ± 3% RH). Our study results serve as a robust foundation for future investigations into the functional roles of *PRX* genes in guava, as well as shedding light on the postharvest dynamics of peroxidase enzyme activity and regulatory genes in this context.

## Results

### Identification, characterization and physico-chemical properties of *PgPRX* genes

A total of 37 *PgPRX* genes were successfully identified within the *P. guajava* genome. These members of the *PgPRX* gene family were systematically named *PgPRX1*-*PgPRX37*, a nomenclature approach grounded in their resemblance to Arabidopsis class-III peroxidase genes. For a comprehensive overview of the gene characteristics, Table 1 provides detailed information regarding gene locations on chromosomes, the lengths of coding sequences (CDS), and the GC content of the *PgPRXs*. Notably, the data reveals that among these *PgPRX* genes, *PgPRX22* (KAI3427825.1) possesses the longest CDS sequence, spanning 1155 base pairs, while *PgPRX33* (KAI3440867.1) boasts the shortest CDS sequence, with a length of 648 base pairs.

**Table 1:** The basic ınformation about *PRX* genes in guava fruit.

Gene name	Chromosome	Start site	End site	Strand	CDS (bp)	GC content (%)
*PgPRX1*	304	247286	247507	−	966	53.8
*PgPRX2*	285	335287	335502	+	990	57.3
*PgPRX3*	283	216045	216176	+	972	50
*PgPRX4*	250	450728	450955	−	1002	57.5
*PgPRX5*	244	445212	445418	+	975	57.3
*PgPRX6*	236	371047	371328	+	1062	57.3
*PgPRX7*	225	408294	408509	+	969	55.5
*PgPRX8*	172	717707	717925	+	963	55.9
*PgPRX9*	157	301252	301479	+	1005	54.5
*PgPRX10*	155	407606	407818	+	978	52.5
*PgPRX11*	136	426980	427183	−	972	52.2
*PgPRX12*	125	557549	557761	+	954	51.9
*PgPRX13*	124	908934	909173	+	990	52.7
*PgPRX14*	121	442229	442435	−	954	56.6
*PgPRX15*	121	865834	866070	+	984	61.4
*PgPRX16*	118	797045	797269	+	978	60.7
*PgPRX17*	116	99959	100183	+	996	50.5
*PgPRX18*	94	314120	314290	+	969	59.3
*PgPRX19*	87	628623	628832	+	978	59.3
*PgPRX20*	81	163748	163831	−	846	47
*PgPRX21*	72	498867	499085	−	969	53.7
*PgPRX22*	72	576865	576906	+	1155	51.3
*PgPRX23*	66	736358	736573	+	984	53.3
*PgPRX24*	59	558918	559121	+	951	54
*PgPRX25*	56	968246	968440	−	984	58.4
*PgPRX26*	43	1043887	1044132	+	978	60.9
*PgPRX27*	40	749283	749480	−	954	53.4
*PgPRX28*	33	1393490	1393897	+	999	56.3
*PgPRX29*	24	584178	584447	+	1041	51.6
*PgPRX30*	23	828231	828437	−	978	57
*PgPRX31*	22	165226	165462	−	1002	54.4
*PgPRX32*	16	108866	109081	−	984	49.2
*PgPRX33*	16	123768	123847	−	648	50.6
*PgPRX34*	15	1535306	1535524	+	993	60.7
*PgPRX35*	15	1539026	1539241	+	990	60.6
*PgPRX36*	4	1763872	1764105	−	1053	54.9
*PgPRX37*	4	2258874	2259149	+	1053	56.1

Detailed information on the specific physico-chemical properties of the PgPRX proteins has been provided in Table 2. Among the 37 PgPRXs, the protein lengths were estimated to range from 215 to 384 amino acids, resulting in molecular weights that varied from a minimum of 23.16 kDa to a maximum of 42.11 kDa. Isoelectric point (pI) values also exhibited considerable variation among the PgPRX proteins. PgPRX20 exhibited the lowest isoelectric point value at 5.12, while PgPRX22 had the highest isoelectric point recorded at 10.16. Furthermore, the analysis of PRX proteins revealed instability index values ranging from 28.94 to 51.33, reflecting varying degrees of protein stability. Additionally, the aliphatic index fell within the range of 71.85–101.20. Intriguingly, the GRAVY (Grand Average of Hydropathy) values for PgPRXs were observed to span from − 0.383 to 0.192, providing insights into their hydrophobic or hydrophilic characteristics (Table 2).

**Table 2:** Physico-chemical properties of PRX proteins in guava fruit.

Gene name	Protein length	MW (Kda)	pI	Instability index	Aliphatic index	GRAVY
*PgPRX1*	321	35.25	9.11	36.01	86.98	− 0.141
*PgPRX2*	329	35.07	8.59	36.51	85.23	0.085
*PgPRX3*	324	35.77	5.58	48.78	82.22	− 0.227
*PgPRX4*	333	35.81	9.31	39.93	80.69	− 0.13
*PgPRX5*	324	34.64	6.36	35.43	74.69	− 0.075
*PgPRX6*	353	38.71	9	42.13	77.68	− 0.224
*PgPRX7*	322	34.1	9.43	35.84	82.73	− 0.093
*PgPRX8*	320	33.48	8.52	40.34	76.91	− 0.061
*PgPRX9*	334	36.61	9.2	44.53	85	− 0.2
*PgPRX10*	325	35.42	5.98	34.78	87.68	− 0.062
*PgPRX11*	323	34.96	8.02	38.57	80.31	− 0.152
*PgPRX12*	318	34.74	5.6	39.1	92.45	0.013
*PgPRX13*	329	35.24	8.82	45.19	77.96	− 0.198
*PgPRX14*	317	34.21	9.29	41.8	88.68	− 0.077
*PgPRX15*	327	36.31	8.91	35.04	82.02	− 0.289
*PgPRX16*	325	34.37	9.42	37.78	101.2	0.192
*PgPRX17*	331	36.31	8.63	51.31	82.57	− 0.195
*PgPRX18*	322	35.02	5.13	36.61	88.2	− 0.079
*PgPRX19*	325	35.2	5.45	42.04	87.29	− 0.027
*PgPRX20*	281	29.81	5.12	29.17	84.38	− 0.11
*PgPRX21*	322	34.43	8.56	30.22	89.13	0.013
*PgPRX22*	384	42.11	10.16	33.89	96.04	0.014
*PgPRX23*	327	35.38	9.02	41.36	74.34	− 0.324
*PgPRX24*	316	33.82	8.6	43.36	89.84	0.03
*PgPRX25*	327	36.39	8.78	32.06	82.08	− 0.157
*PgPRX26*	325	35.43	6.44	38.4	71.85	− 0.147
*PgPRX27*	317	33.87	5.85	41.02	86.15	0.048
*PgPRX28*	332	35.55	8.91	43.33	76.72	− 0.121
*PgPRX29*	346	37.37	6.13	41.19	90.2	− 0.06
*PgPRX30*	325	35.51	9.69	40.21	81.11	− 0.123
*PgPRX31*	333	37.41	7.64	37.94	89.64	− 0.383
*PgPRX32*	327	35.42	7.57	40.25	85.66	− 0.089
*PgPRX33*	215	23.16	9.43	28.94	83.91	− 0.149
*PgPRX34*	330	35.63	8.89	34.73	78.7	− 0.128
*PgPRX35*	329	35.63	9.33	30.2	78.02	− 0.131
*PgPRX36*	350	38.23	5.53	40.57	90.8	− 0.116
*PgPRX37*	350	37.88	8.6	42.98	86.94	− 0.041

### Conserved domain and motif analysis of *PgPRX*s

**Figure 1:**
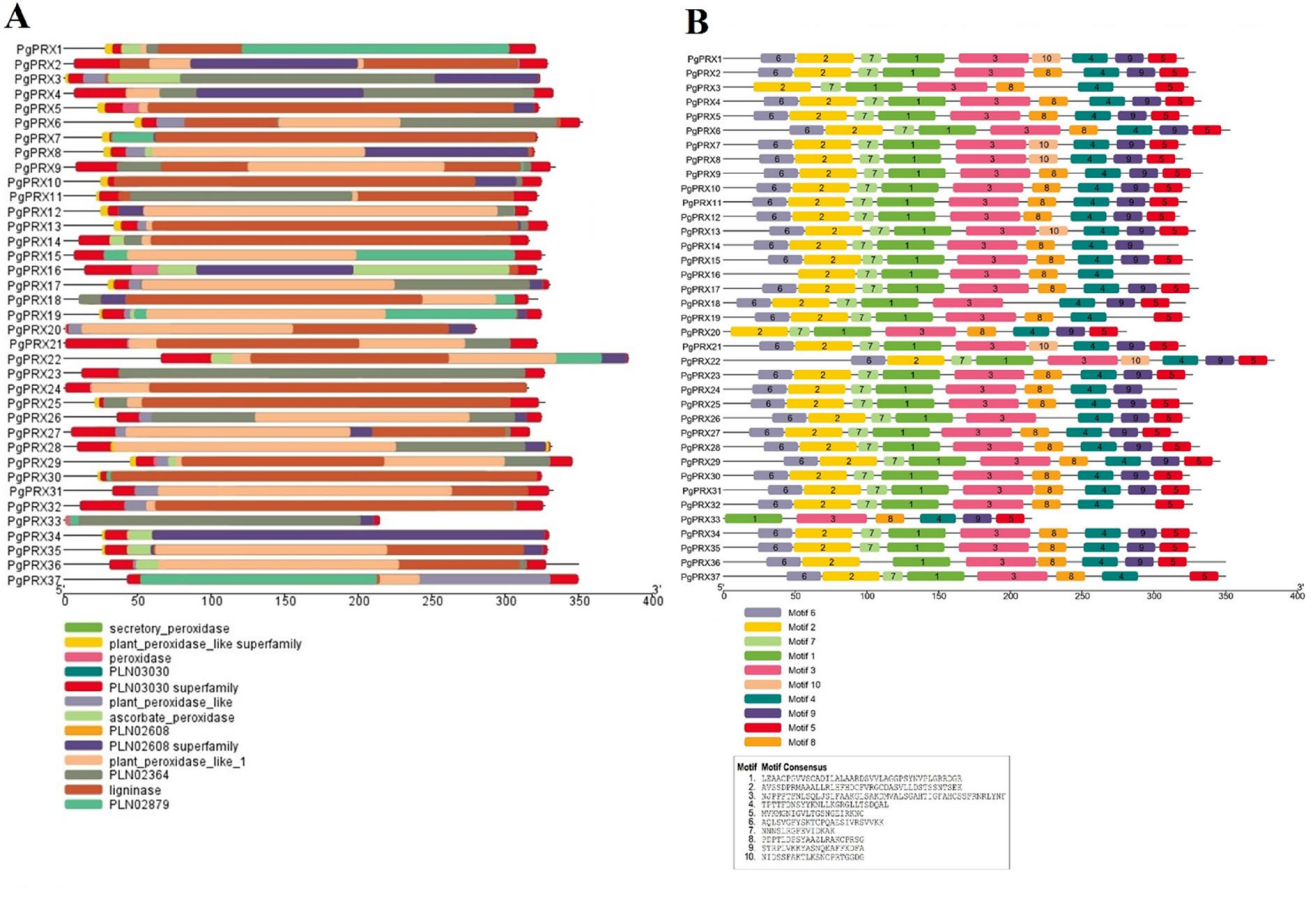
Conserved domain (**A**) and motif analysis (**B**) of guava class-III peroxidase genes.

The analysis of conserved domains revealed that every PgPRX protein was found to contain at least one peroxidase domain (cl00196), as illustrated in Fig. 1A. To further delve into the conservation patterns, we employed online MEME servers to investigate the distribution of conserved motifs among PgPRXs. This investigation unveiled a range of 6–10 presumed conserved motifs present across the PgPRX proteins (Fig. 1B).

Comparison with Fig. S1 reveals a striking similarity in the organization and composition of motifs among PgPRXs within all three phylogenetic groups. This observation strongly suggests that throughout the course of evolution, PgPRXs have exhibited a remarkable level of conservation in their motif arrangements and compositions.

### Subcellular localization of PgPRX proteins

Numerous PgPRX proteins are found in different subcellular locations, with specific proteins displaying strong representation within certain organelles or cellular compartments. Notable localizations include the presence of several PgPRX proteins in the cytoskeleton, nucleus, cytoplasmic skeleton, extracellular region, endoplasmic reticulum, mitochondria, peroxisomes, chloroplasts, plasma membrane, Golgi apparatus, and vacuoles. Several PgPRX proteins are mainly localized to the chloroplast, plasma membrane, vacuole and extracellular regions of the plant cell (Fig. S2).

### Chromosomal mapping and syntenic analysis of *PgPRX* genes

**Figure 2:**
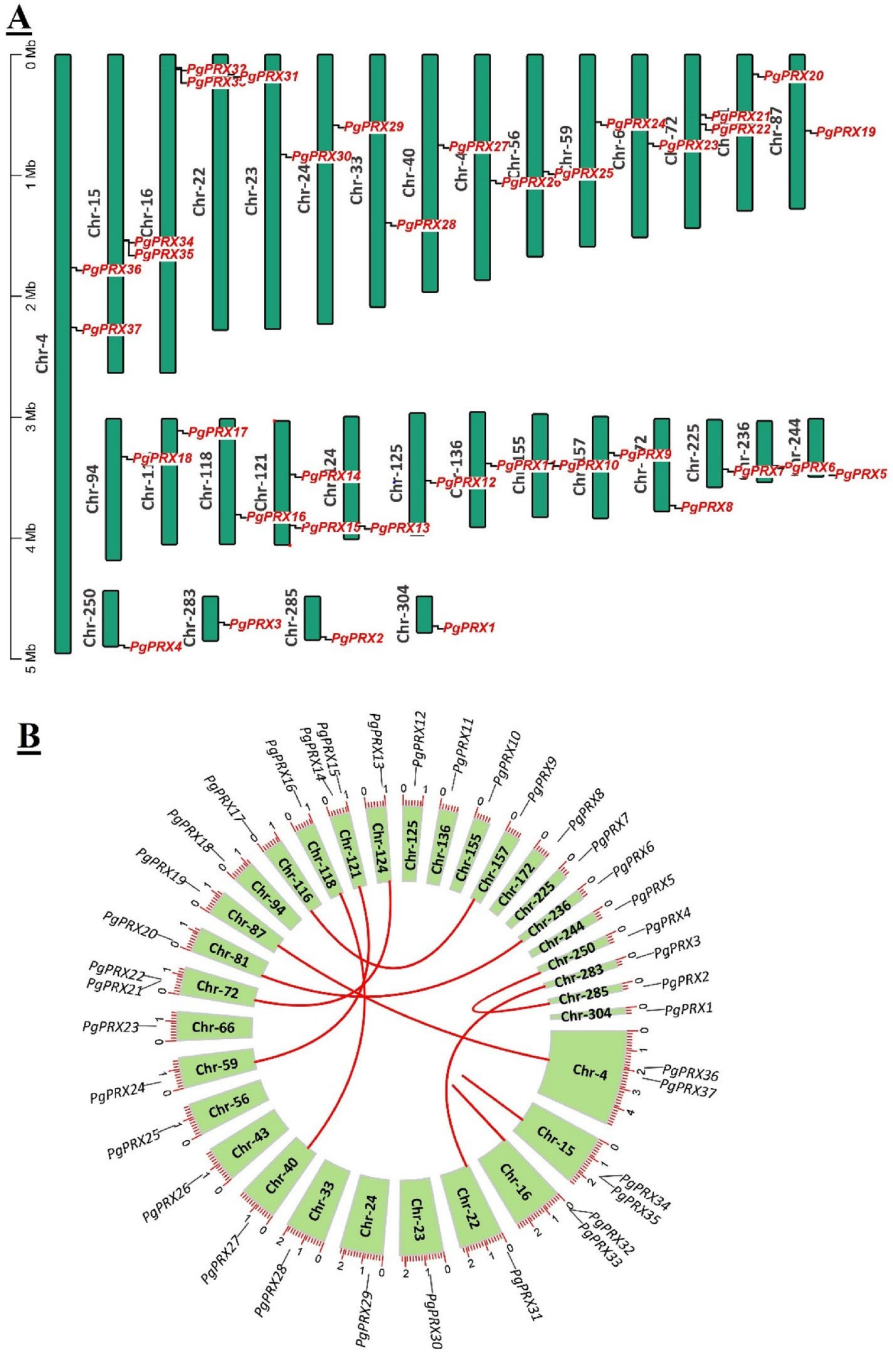
Chromosomal mapping and gene duplication of *PgPRXs*.

The chromosomal mapping of the 37 *PgPRXs* has been illustrated in Fig. 2. These genes have been mapped onto various scaffold chromosomes, with only the scaffold chromosomes displayed in Fig. 2. Notably, four chromosomes, namely Chr-16, Chr-15, Chr-4, and Chr-72, each contained 2 *PgPRXs*, as depicted in Fig. 2A.

Furthermore, when conducting syntenic analysis of the *PgPRXs* genes, it was observed that there were only two pairs of tandemly repeated sequences, specifically *PgPRX32*–*PgPRX33* and *PgPRX34*–*PgPRX35*, among the 37 *PgPRX* genes, as depicted in Fig. 2B.

In contrast, the predominant duplication pattern was identified as "whole-genome duplication (WGD) or segmental duplication." Eight pairs of WGD-repeated genes were identified: *PgPRX2*–*PgPRX4*, *PgPRX3*–*PgPRX31*, *PgPRX6*–*PgPRX20*, *PgPRX9*–*PgPRX17*, *PgPRX13*–*PgPRX21*, *PgPRX14*–*PgPRX24*, *PgPRX16*–*PgPRX27*, and *PgPRX19*–*PgPRX37*. This insight provides valuable information on the evolutionary history of the *PgPRX* gene family.

To further analyze whether these tandem or segmental repeated genes are under selection pressure during evolution, we calculated the K_a_ and K_s_ values of these genes^32^. The analysis results show that the K_a_/K_s_ values of these *PgPRXs* sequences are less than 1 (Table 3), indicating that these genes have been gone through purified selection during evolution process. However, 2 pairs i.e., *PgPRX6*–*PgPRX20* and *PgPRX16*–*PgPRX27* exhibited high sequence divergence value (pS ≥ 0.75).

**Table 3:** The K_a_ / K_s_ ratio of duplicated *PePOD* genes.

Gene 1	Gene 2	K_a_	K_s_	K_a_/K_s_	Selection	Duplication
*PgPRX2*	*PgPRX4*	0.081400	0.386832	0.210428	Purifying	Segmental/WGD
*PgPRX3*	*PgPRX31*	0.673283	5.023084	0.134038	Purifying	Segmental/WGD
*PgPRX6*	*PgPRX20*	0.599564	−	−	–	Segmental/WGD
*PgPRX9*	*PgPRX17*	0.186074	1.744935	0.106637	Purifying	Segmental/WGD
*PgPRX13*	*PgPRX21*	0.189801	1.159416	0.163704	Purifying	Segmental/WGD
*PgPRX14*	*PgPRX24*	0.173722	1.023366	0.169756	Purifying	Segmental/WGD
*PgPRX16*	*PgPRX27*	0.440267	−	−	–	Segmental/WGD
*PgPRX19*	*PgPRX37*	0.307599	1.458193	0.210945	Purifying	Segmental/WGD
*PgPRX32*	*PgPRX33*	0.407264	1.950443	0.208806	Purifying	Tandem
*PgPRX34*	*PgPRX35*	0.053607	0.272597	0.196651	Purifying	Tandem

### Phylogenetic relationship between PRX proteins of *Psidium guajava* and *Arabidopsis thaliana*

**Figure 3:**
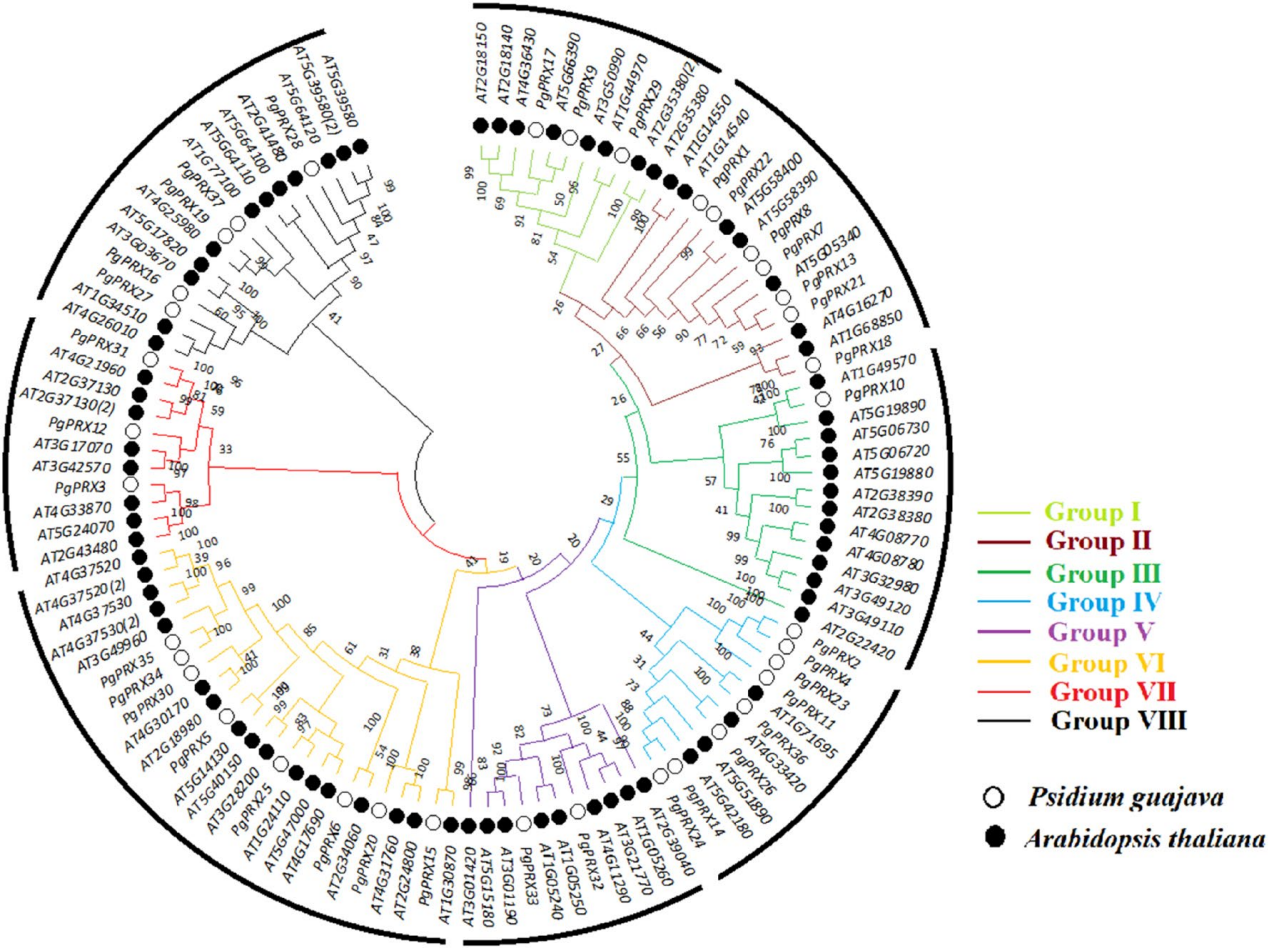
Phylogenetic relationships between Class-III peroxidase genes in *Psidium guajava* and *Arabidopsis thaliana*. Distinct phylogenetic groups are highlighted with differently colored connecting lines.

The phylogenetic tree of PgPRXs and AtPRXs was generated using MEGA-X. These proteins were classified into eight distinct groups (I–VIII) based on their sequence similarity, as depicted in Fig. 3. Remarkably, all 37 PgPRXs were distributed across these eight groups, with group I, II, III, IV, V, VI, VII, and VIII accommodating three, seven, one, eight, two, eight, three, and five genes, respectively.

The grouping outcomes obtained from the comprehensive phylogenetic tree were generally in accordance with those derived from the phylogenetic tree constructed using the PgPRX protein sequences. Furthermore, when analyzing the results of conserved motifs, it was evident that members of the same subgroup exhibited a comparable number and type of conserved motifs. This alignment between conserved motifs and phylogenetic grouping further reinforces the reliability of the phylogenetic tree results (Fig. 3).

### Promoter region analysis of *PgPRXs*

**Figure 4:**
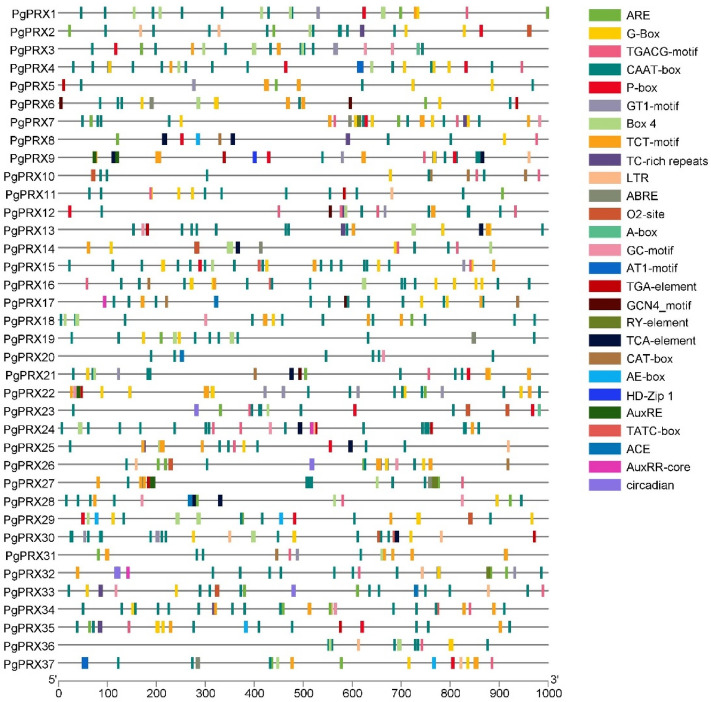
Cis-regulatory elements detected in the promoter sequences of *PgPRX* genes.

To further delve into the transcriptional mechanisms underlying *PgPRXs*, we conducted a promoter analysis, focusing on a 1000-base-pair segment upstream of the peroxidase genes (Fig. 4). Within these promoter regions, we identified several cis-elements associated with plant growth hormones, including ABRE, AuxRR-core, AuxRE, TATC-Box, P-Box, GARE, TCA-element, TGACG-motif, and TGA-element. These cis-elements are responsible for regulating the response to abscisic acid, auxins, methyl jasmonate, gibberellins, and salicylic acid.

In addition to these, we also detected stress-responsive cis-elements such as LTR and TC-rich repeats in several genes. Furthermore, we found light-responsive cis-elements like the G-Box, GT1-motif, Box 4, TCT-motif, ACE, and AE-box. Anaerobic induction-related cis-regulatory elements e.g., ARE and GC-motif were also detected. Beyond these, *PgPRX* genes were shown to contain O2-site, GCN4-motif, RY element, HD-Zip1, and circadian cis-elements, each playing a distinct role in zein metabolism regulation, endosperm expression, seed-specific regulation, differentiation of the palisade mesophyll cells, and circadian control, respectively. The details of detected cis-elements have been provided in Table S1.

### Protein–protein interactions among PgPRXs

**Figure 5:**
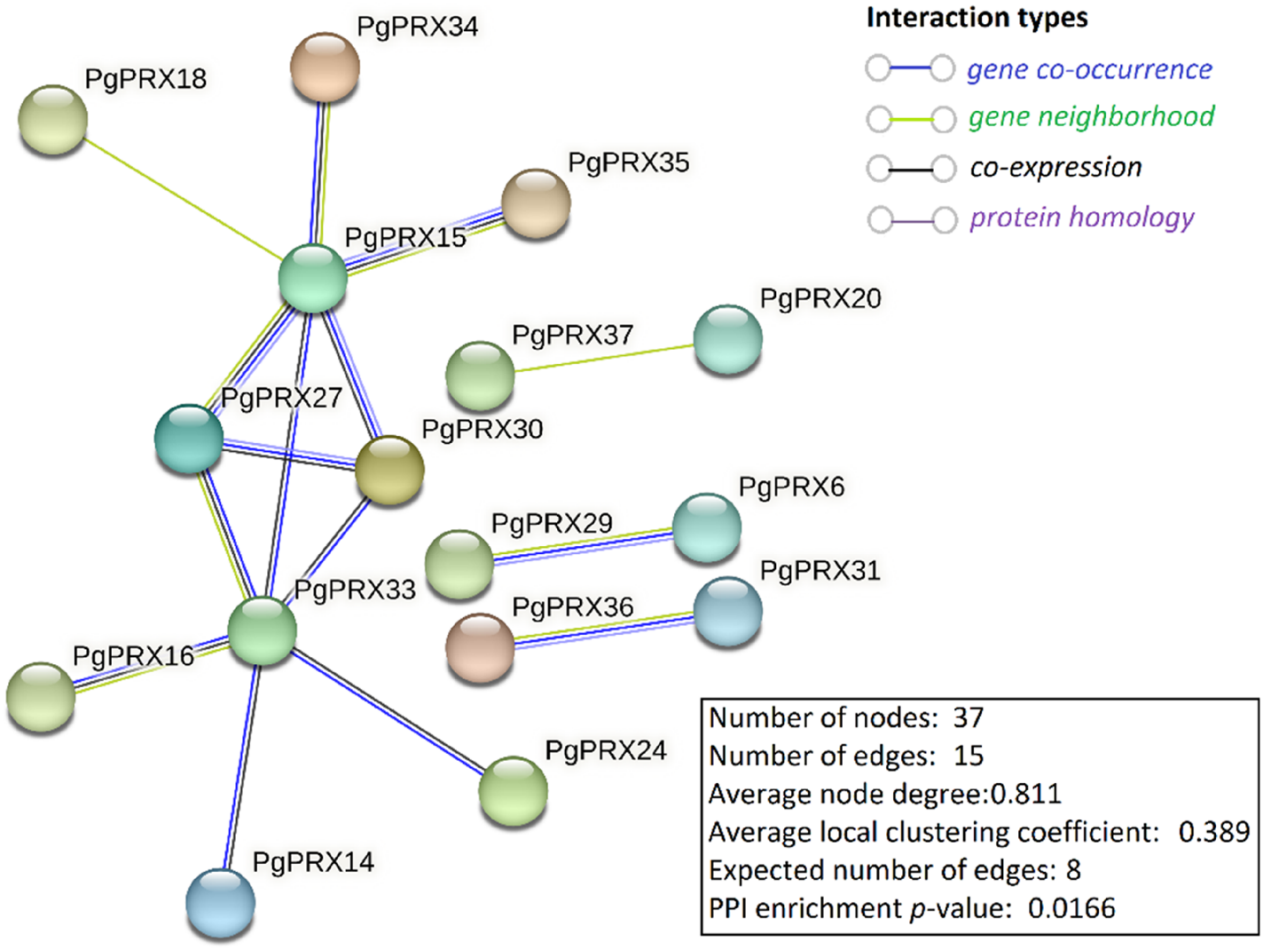
Predicted protein–protein interactions of PgPRXs visualized by STRING. The nodes indicate proteins, and edges indicate the number of interactions. The number of average interactions per node is indicated by the node degree. The clustering coefficient specifies the average node density of the map. Disconnected nodes are hidden, and only interactions with a confidence score of ≥ 0.4 are shown.

Protein–protein interaction networking predictions among PgPRXs were conducted. The analysis revealed a robust interaction network involving ten PgPRX proteins: PgPRX14, PgPRX15, PgPRX16, PgPRX18, PgPRX24, PgPRX27, PgPRX30, PgPRX33, PgPRX34, and PgPRX35. Core interactions were identified, with PgPRX15 and PgPRX33 acting as central proteins interacting with the remaining eight PgPRX proteins. PgPRX27 and PgPRX30 displayed interactions with three PgPRX proteins each. Additionally, specific point-to-point interactions were observed, such as those between PgPRX20 and PgPRX37, PgPRX6 and PgPRX29, as well as PgPRX31 and PgPRX36 (Fig. 5).

### Genetic expressions of *PgPRXs* in different tissues of guava

**Figure 6:**
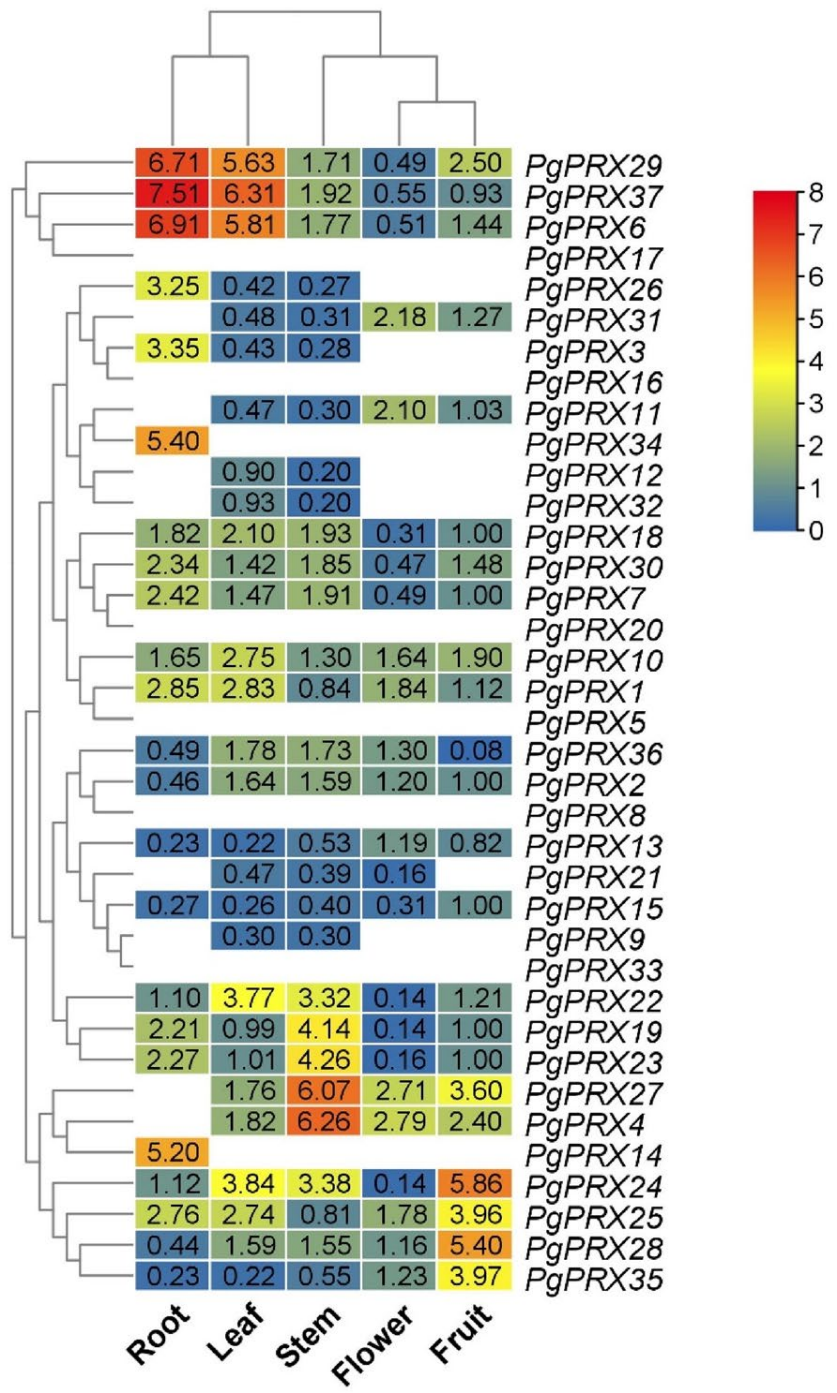
Expression patterns of PgPRXs in various guava plant tissues, including roots, mature leaves, stems, full-bloom flowers, and ripened fruits, were analyzed, and a heatmap was generated based on relative expression levels. In the heatmap, the color scheme is as follows: red indicates higher expression, blue indicates lower expression, and white represents no detectable expression.

Furthermore, our investigation extended to the examination of the relative gene expression within the peroxidase gene family across various guava tissues, specifically mature leaves, roots, stems, full-bloom flowers, and ripened fruits (Fig. 6). The expression patterns of guava *PRXs* displayed significant variations among these different plant tissues.

Among the 37 *PgPRXs*, we observed that the transcript levels of 13 genes were relatively low in the examined tissues, with expression levels ranging from 0 to 2. On the other hand, five *PgPRXs*, namely *PgPRX6*, *PgPRX14*, *PgPRX29*, *PgPRX34*, and *PgPRX37*, exhibited notably higher expression levels in guava roots, exceeding 5. Similarly, *PgPRX6*, *PgPRX22*, *PgPRX24*, *PgPRX29*, and *PgPRX37* showed elevated expressions in guava leaves, with levels ranging from 3.77 to 6.31. In stem tissues of guava, *PgPRX19*, *PgPRX22*, *PgPRX23*, and *PgPRX24* demonstrated high levels of expression.

Notably, four genes, specifically *PgPRX4*, *PgPRX11*, *PgPRX27*, and *PgPRX31*, displayed increased expressions exceeding 2 in full-bloom flowers. In guava fruits, seven genes, including *PgPRX4*, *PgPRX24*, *PgPRX25*, *PgPRX27*, *PgPRX28*, *PgPRX29*, and *PgPRX35*, exhibited relative expressions surpassing 2.

### Peroxidase activity and expression profiling of *PgPRX* genes in guava fruits stored at two different temperatures

**Figure 7:**
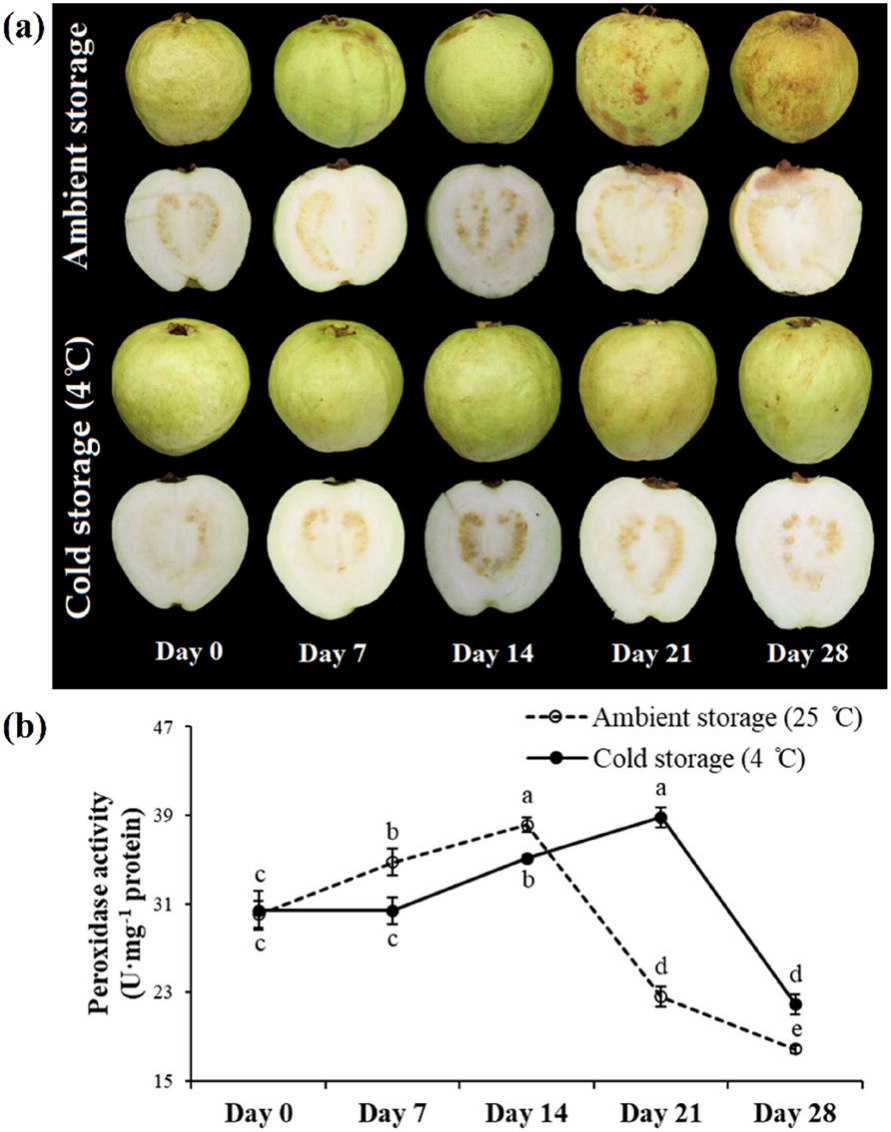
Fruit visual appearance and peroxidase activity in guava fruits stored under two different conditions: ambient storage at 25 ± 2 °C and cold storage at 4 ± 1 °C, both for a period of 28 days. Same letters in line chart indicate non-significant (*p* ≤ 0.05) difference between treatment variables, according to Fisher’s LSD technique.

The visual transformations of guava fruit during both ambient and cold storage are represented in Fig. 7a. The results showed a gradual yet statistically significant (*p* ≤ 0.05) increase in peroxidase activity during the postharvest storage period. The highest levels were observed at 14 days of ambient storage (38.13 U mg^−1^ protein) and 21 days of cold storage (38.81 U mg^−1^ protein). Nevertheless, peroxidase activity decreased to its minimum levels after the 14th day of ambient storage and the 21st day of cold storage, irrespective of the storage conditions (Fig. 7b).

**Figure 8:**
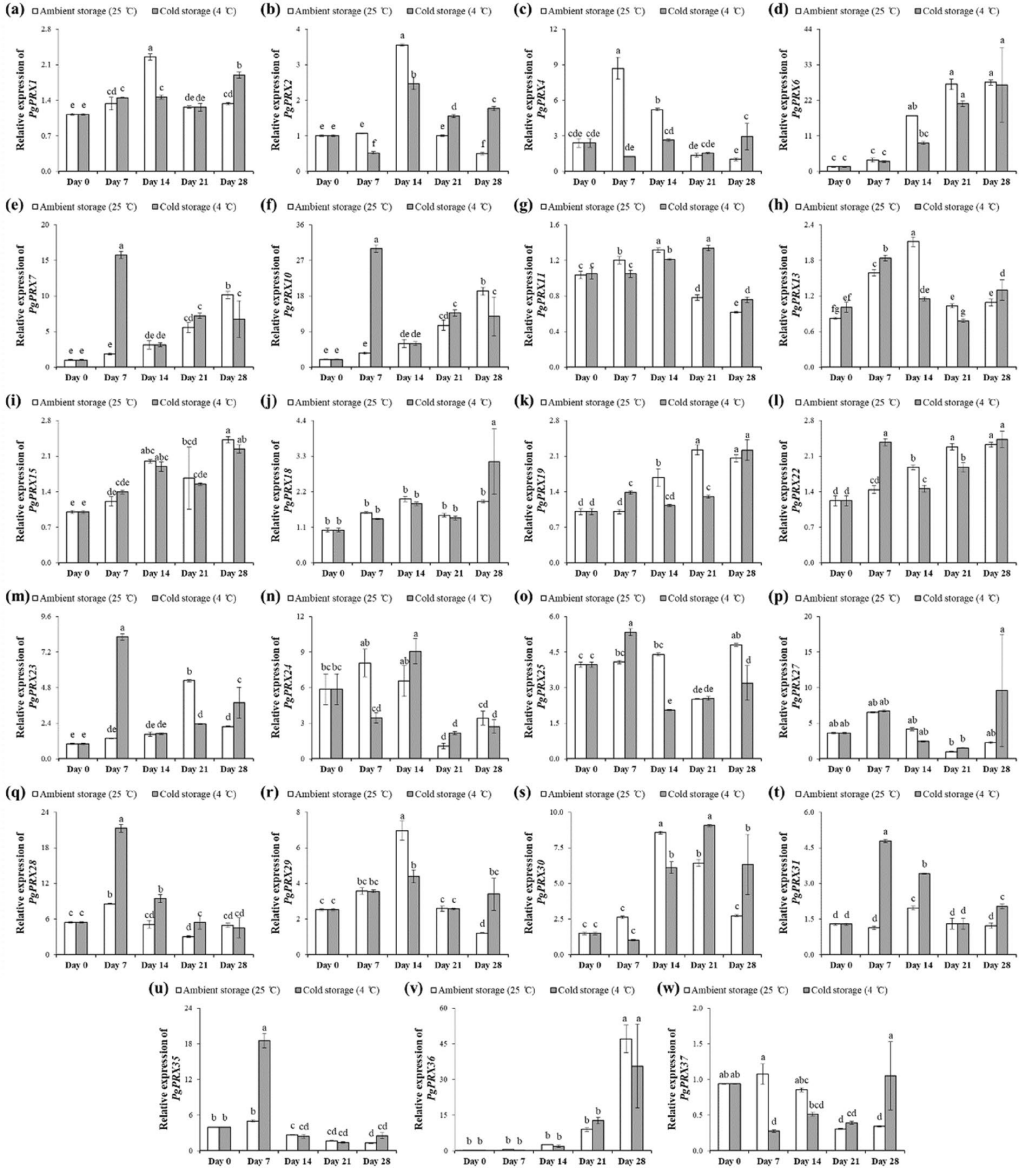
The relative expressions of *PgPRX* genes in guava fruits stored under two different conditions: ambient storage at 25 ± 2 °C and cold storage at 4 ± 1 °C, both for a period of 28 days. Each treatment had three biological and three technical replications. Same letters bar charts indicate non-significant (*p* ≤ 0.05) difference between treatment variables, according to Fisher’s LSD technique.

Postharvest storage conditions had a significant impact on the expression of *PgPRX* genes in guava fruits (Fig. 8). Out of the 37 *PgPRXs* studied, 23 were found to be expressed, while 14 genes did not show any expression. Among the 23 expressed *PgPRXs*, eight specific genes, namely *PgPRX1*, *PgPRX2*, *PgPRX11*, *PgPRX13*, *PgPRX15*, *PgPRX29*, *PgPRX30*, and *PgPRX37*, exhibited their highest expression levels on the 14th day of ambient storage.

In the case of *PgPRX4*, cold storage led to a significant downregulation of expression on the 7th and 14th days of postharvest storage when compared to ambient storage. On the contrary, the expression of *PgPRX6* increased with an extended storage period, regardless of the storage conditions.

The expression patterns of *PgPRX7* and *PgPRX10* were almost identical, with the expression of these genes significantly upregulated under the influence of cold storage. Notably, six *PgPRX* genes, including *PgPRX18*, *PgPRX19*, *PgPRX22*, *PgPRX27*, *PgPRX36*, and *PgPRX37*, displayed their maximum expression levels on the 28^th^ day of cold storage. In the cases of *PgPRX19*, *PgPRX27*, and *PgPRX37*, their expressions were significantly different from those observed in ambient-stored fruits.

Five genes, *PgPRX23*, *PgPRX25*, *PgPRX28*, *PgPRX31*, and *PgPRX35*, exhibited their highest expressions in cold-stored fruits on the 7th day of storage, and these levels were significantly different from those in ambient-stored fruits.

## Discussion

Peroxidases play a pivotal role in regulating growth and the life cycle of plants. In addition to their involvement in growth regulation, extensive research has been conducted on the role of *PRX* genes in plant stress responses^33,34^. Genome-wide studies and the characterization of the *PRX* gene family have been undertaken in various species and model plants, including Arabidopsis, rice, pear, and passion fruit^6,11,24,26^.

In our study, we identified a total of 37 *PRX* genes in guava, which is fewer than the 73 *PRXs* found in Arabidopsis. Our phylogenetic analysis revealed closely related members of *PRXs* between *A. thaliana* and *P. guajava*. Each cluster contained *PRX* members from guava, even though their number was relatively lower (as shown in Fig. 3). Furthermore, we found that 12 out of the 37 *PgPRX* genes formed ortholog pairs with Arabidopsis *PRXs*, indicating a specific expansion of the *PRX* gene family, a pattern consistent with findings reported by Yang et al.^8^ and also observed in passion fruit by Liang et al.^26^.

Additionally, when we examined the K_a_/K_s_ values of duplicated *PgPRX* genes (Table 3), we found that they were less than 1. This suggests that the *PRX* gene family underwent significant selective pressure following the domestication of guava^35,36^. When examining the protein domain structure and motif organization of PgPRX proteins, we observed a phenomenon similar to that found in other species such as rice and maize^11,21^. This phenomenon indicates that proteins belonging to the same subgroups (as depicted in Fig. 1) exhibit similar structural compositions in terms of their domain architecture and motif constitution. The domain structure of a protein represents its fundamental configuration. These findings suggest that PgPRXs with similar structural compositions likely share functional similarities, in accordance with the observations made by Liang et al.^26^.

The terms "tandem" and "segmental/WGD duplication" play a crucial role in the expansion of gene families^37^. In response to selective evolution, gene duplication events can theoretically lead to the emergence of two gene copies with either similar or novel functional characteristics. This often results in the expansion of a gene family^37,38^. Tandem duplication events typically impact a limited number of genes, usually those in close proximity to each other, and result from unequal and multiple crossing-over events^39^. On the other hand, segmental duplication, often called as whole-genome duplication (WGD), involves larger fragmental duplications in the genome and may give rise to gene copies with numerous rearrangements and deletions^40^. In our study of the guava *PRX* gene family, we identified a total of 10 duplicated gene pairs, comprising 8 segmental duplication pairs and 2 tandem duplicated pairs (Fig. 2, Table 3). Notably, the percentage of duplicated gene pairs in guava was higher than what was observed in rice and maize^11,21^. In guava, the gene pairs resulting from segmental duplication were more prevalent than those arising from tandem duplication, indicating that the rate of segmental duplication played a dominant role in the expansion of the *PgPRX* gene family.

The expression profiles of related genes offer valuable insights into their roles within the plant. In the case of the 37 *PgPRXs*, their relative gene expression exhibited distinctive and dynamic profiles across various guava tissues. Notably, 13 of these genes displayed either weak expression or no expression at all in fruit tissues, as illustrated in Fig. 6. This observation strongly implies the existence of functional diversity among PgPRX proteins within the guava plant.

During the postharvest storage of guava fruits, changes in peroxidase levels and their gene expressions are of particular interest in understanding fruit quality and shelf-life maintenance. In current study, the expressions of different *PgPRX* genes exhibited distinct patterns during storage, with some genes showing increased expression over time, while others were downregulated, depending on the storage conditions (ambient or cold storage). For instance, *PgPRX1*, *PgPRX2*, *PgPRX11*, *PgPRX13*, *PgPRX15*, *PgPRX29*, *PgPRX30*, and *PgPRX37* displayed their maximum expression at the 14th day of ambient storage (Fig. 8), indicating a potential role in maintaining fruit quality during this period. Conversely, *PgPRX4* had reduced expression during cold storage, possibly affecting postharvest quality of guava fruits. The *PgPRX15*, *PgPRX27*, *PgPRX30*, *PgPRX33* genes were predicted to play crucial roles in the interaction network of PgPRX proteins (Fig. 5) and could coordinate important crosstalk between defense responses to microbial infection and postharvest decay in guava. Such insights into peroxidase gene expression dynamics provide valuable information for the postharvest management of guava fruits, contributing to enhanced fruit quality and extended shelf life.

## Materials and methods

### Identification, physico-chemical properties, and subcellular localization of *PgPRX* genes

To identify *PRX* genes in guava, we conducted a search within the *Psidium guajava* Genome Project available at the National Center for Biotechnology Information (NCBI) (https://www.ncbi.nlm.nih.gov/datasets/genome/GCA_023344035.1/)^31^. For phylogenetic analysis, genome sequences of *Arabidopsis thaliana* were obtained from the Arabidopsis Information Resource (TAIR) (http://www.arabidopsis.org/). We used Arabidopsis *PRX* genes as query sequences to perform BLAST searches against the guava genome database. Additionally, we employed the HMMER3 software package to retrieve the seed alignment file for the peroxidase domain (CL0617) from the Pfam database^41^. Subsequently, the HMMER software suite was used to conduct HMM searches against the local guava protein database^42^.

We further assessed the physical locations of all potential *PRX* genes and excluded redundant sequence repeats located on the same chromosome. Additionally, all retrieved PRX protein sequences were subjected to re-analysis using the SMART programs from the Pfam database (http://smart.embl-heidelberg.de) to confirm the presence of the peroxidase domain. Any protein sequences lacking this domain were excluded from the study.

The physicochemical characteristics of PgPRX proteins were determined using the ExPASy Proteomics Server (http://web.expasy.org/compute_pi/). Furthermore, we predicted the subcellular localization of PgPRX proteins by utilizing the WoLF PSORT web server (https://wolfpsort.hgc.jp/).

### Conserved motif analysis of *PgPRX* genes

We employed the MEME suite server (http://meme-suite.org/) to identify conserved motifs in the sequences of *PRX* genes. The analysis was performed with the following parameter settings: a maximum of 10 different motifs, a minimum motif width of 10, and a maximum motif width of 50.

### Chromosomal mapping and syntenic analysis of *PgPRX*

For assessing the distribution and mapping of *PgPRX* genes in the *P. guajava* genome, we utilized a gff3-file and the software Tbtools (version 0.6655)^43^. To identify duplicated *PRX* genes within the guava genome, we employed the MCScanX software, following the methodology outlined by Wang et al. (2012).

To identify syntenic gene pairs and types of duplication, we conducted BLASTP comparisons of all PRX protein sequences from guava. This was done using the BLASTP tool available at http://www.ncbi.nlm.nih.gov/blast/blast.cgi, with an *e*-value threshold of less than 1 × 10^−5^. The BLASTP outputs, along with gene-location files, were processed with default settings as input data for MCScanX.

### K_a_ and K_s_ calculation

We calculated the K_a_ (nonsynonymous) and K_s_ (synonymous) values for syntenic gene pairs using the downstream analysis tools in MCScanX. Specifically, we employed the KaKs_Calculator (version 2.0) and utilized the Nei–Gojobori (NG) method^44^.

### Phylogenetic analysis

To conduct phylogenetic and molecular evolutionary genetics studies, we employed Molecular Evolutionary Genetics Analysis X (MEGA-X, version 10.2.6)^45^. Multiple sequence alignment was carried out using MEGA-X with default settings, utilizing the Multiple Sequence Comparison by Log-Expectation (MUSCLE) method.

To generate various PRX trees, the neighbor-joining (NJ) approach was applied with a bootstrap analysis consisting of 1000 replicates, employing the *p*-distance method and pairwise deletion. These analyses were also executed using MEGA-X.

### Promoter region analysis and protein–protein interaction

Protein–protein interaction networking of *PgPRX* family genes was predicted using the STRING database (http://www.string-db.org). The PlantCARE database was used to analyze cis-regulatory elements for each gene analyzed beginning from the start codon to 1 kb upstream (https://bioinformatics.psb.ugent.be/webtools/plantcare/html/).

### Plant material and treatments

In late May 2023, samples were collected from various guava tissues (Cv. Baixin) obtained from a commercial orchard. These included full-bloom flowers, roots, mature leaves, ripened fruits, and stems, all intended for real-time quantitative PCR (RT-qPCR) analysis.

For postharvest storage study, mature guava fruits of same cultivar were harvested from the same orchard and transported to the laboratory. The guava fruits were carefully sorted to ensure uniformity in terms of color, size, and maturity stage. Subsequently, they underwent a thorough cleaning process, including washing and disinfection, achieved by immersing them in a 0.01% sodium hypochlorite (NaClO) solution for 3 min, followed by a rinse. After this preparation, the fruits were air-dried at a controlled temperature of 25 ± 2 °C for a duration of 90 min.

The prepared fruits were then stored under two different conditions: ambient storage at 25 ± 2 °C (40–60% RH) and cold storage at 4 ± 1 °C (85 ± 3% RH), both for a period of 28 days. At specific intervals (0, 7, 14, 21, and 28 days), samples from the stored fruits were collected and rapidly frozen using liquid nitrogen (− 196 °C). These frozen samples were subsequently stored in an ultra-low-temperature refrigerator (− 80 °C) for further enzymatic and RT-qPCR analysis. It's worth noting that each treatment was replicated three times for biological and technical consistency. Each replicate consisted of 5 guava fruits.

### Peroxidase enzyme activity assay, RNA isolation and quantitative RT-PCR analysis

Peroxidase activity was assessed following a well-established method as detailed in the work by Hasanuzzaman et al.^46^. The change in absorbance was monitored at a wavelength of 470 nm over a 4-min period.

For RNA isolation, guava samples were processed using the Total RNA kit from TianGen Biotech in Beijing, China. The quantity and quality of the isolated RNA were determined using a NanoDrop N-1000 spectrophotometer from NanoDrop Technologies in Wilmington, DE, USA, as well as agarose gel electrophoresis.

To generate first-strand cDNA, 1 µg of RNA was employed with the Prime Script RT Reagent Kit featuring a gDNA Eraser from TaKaRa in Dalian, China. RT-qPCR analysis was performed using a high-performance real-time PCR instrument (LightCycler® 96) from Roche Applied Science in Penzberg, Germany.

The 2^−ΔCT^ method was employed to calculate the relative expressions of *PgPRX* genes, with each analysis consisting of 3 biological replicates and 3 technical replicates. As an internal control, the actin protein, which has been previously documented^47,48^ was selected. A complete list of the primers utilized for RT-qPCR can be found in Table 4.

**Table 4:** Primer sequences of *PePOD* genes.

Gene name	Gene IDs	Forward primer (5ʹ–3ʹ)	Reverse primer (5ʹ–3ʹ)
*PgPRX1*	KAI3408259.1	GACTACGTCTGCCCTCAAGC	TCAACCTCCGCCTTAATTTG
*PgPRX2*	KAI3408630.1	GCATTTCCCACTGTTCTTCC	GGAGCTTCGACAACTGAAGG
*PgPRX3*	KAI3408725.1	GAAACCGAAGCCATTCCATA	CTCCTGCCAGTGAAAAGAGG
*PgPRX4*	KAI3409548.1	GAGTCTCCCACTGCTCTTCG	GGAGCTTCGACAACTGAAGG
*PgPRX5*	KAI3409738.1	AGACGGTGACCACCAAGTTC	ACAGTGTCAAACCCGTCTCC
*PgPRX6*	KAI3410582.1	GGTGGAAGGATTCGAGACAA	TGTTAGGTGGCACTCTCGTG
*PgPRX7*	KAI3410816.1	CCTGGACGATACATCCTCGT	TGCGTCTCTTCTTCCCAGTT
*PgPRX8*	KAI3414506.1	AAAGGCTTCACTGCCAAAGA	GAAGTAGGCGTTGTCGAAGG
*PgPRX9*	KAI3415119.1	CTTGTTGCCCTCTCTGGAAG	TTATCGGGCTGACAAAGTCC
*PgPRX10*	KAI3416602.1	ATTTTATGCAGTGCCCTTGG	TGCGCACCTGATAGCACTAC
*PgPRX11*	KAI3417851.1	TGACCCGAACCTAGTTGTCC	GCCGACTTTACCCATCTTCA
*PgPRX12*	KAI3419279.1	ACTGCCAAGTACAGGGATGC	GCAATGTACGGTCCTCCACT
*PgPRX13*	KAI3419519.1	GAATGTGAAACTCGGGAGGA	AAGGAGGAGTCCAGGTTGGT
*PgPRX14*	KAI3419837.1	AACTCGACGGCAAAGAACAC	TTCTTCCTTTTGGCACATCC
*PgPRX15*	KAI3419887.1	TGAGCTCTTCCAATCCCAAG	CTTCCGCTTCAGGTAGTTCG
*PgPRX16*	KAI3420548.1	AGTTCCACGATTGCTTCGTC	TAATGTCAGCGCAAGAAACG
*PgPRX17*	KAI3420628.1	GGACTCGAGAAGTGCCAGTC	AAGTGTGGAGTCCGGTTGAC
*PgPRX18*	KAI3423458.1	GATCGTGAGGAAGGAGATGG	TTCGAGCATGTTCTTGATGC
*PgPRX19*	KAI3424655.1	CATCCAACAGCTCAAGTCCA	GATTGCTATCCGCACATTCA
*PgPRX20*	KAI3426523.1	CTGCAGTTCAGATTCCGACA	AGCCGCTCCTATGGTATGTG
*PgPRX21*	KAI3427823.1	GGATCGAACCTCTTGGATGA	TCTCCTTCCGAGCTTCACAT
*PgPRX22*	KAI3427825.1	AAGTTGCGGATGAAATGTCC	AAATCCCGGAAAAATGCTCT
*PgPRX23*	KAI3430055.1	CCTCTTTCGGCTTCTGTGTC	GAATCCAGCAAGATCGAAGC
*PgPRX24*	KAI3431993.1	GAAGAAGGCAGTGGAAGCAC	GTTGAAGGTGGGAGCTGGTA
*PgPRX25*	KAI3433098.1	CATTCAACGACGTGATGACC	TCCCCGTCTTCACCTGATAC
*PgPRX26*	KAI3435434.1	GATGCTGTTGATGGAGACGA	CTGAATGAAGCAGTCGTGGA
*PgPRX27*	KAI3437042.1	ACTGCAGCTTCTTCCGAGAC	GTTGTCAACGATGGACGATG
*PgPRX28*	KAI3439285.1	TGCACTTCCATGACTGCTTC	GCGAGAGCGAGAATATCAGC
*PgPRX29*	KAI3440041.1	TCCTCTTGGAAGGAGGGACT	GCTGTTCCCATTCTGGTTGT
*PgPRX30*	KAI3440110.1	CGAACGTCGAGTCCCTAGTC	TCCTGCACTGAGGAACACTG
*PgPRX31*	KAI3440147.1	CTCGACGCCATTAAAGAAGC	TCCGGAAGGTATTGTTCGAG
*PgPRX32*	KAI3440865.1	TCAGCCGCTCTACTGAGGAT	TGCACAAGAGACGATTCCAG
*PgPRX33*	KAI3440867.1	TTCTTGCCCTAGTTGCTCGT	TTTGACGGTAAGACCCTTCG
*PgPRX34*	KAI3441017.1	CAAGCTGACATGATCGCACT	GTCCATGTCGATGGCTCTCT
*PgPRX35*	KAI3441018.1	AGCTCAATGCCTTGTTTGCT	GTCCATGTTTATGGCGATCC
*PgPRX36*	KAI3442621.1	TTCCCGTTTCAGTTCTTTCG	CTGAACAAAGCAGTCGTGGA
*PgPRX37*	KAI3442665.1	GATCGAAAATGGGGATAGCA	GCTCAAGAAAACCGCATCTC
*PgACT*	KAI3439588.1	TCCATCATGAAGTGCGATGT	ATTCTGCCTTTGCAATCCAC

### Ethical declarations

This study was complied with the relevant institutional, national, and international guidelines and legislations. Relevant permits/permissions/licences were obtained to collect plant parts of guava (Cv. Baixin) from a commercial orchard situated in Fuzhou city, Fujian.

## Conclusions

In this study, we conducted a comprehensive analysis of the *PRX* gene family in guava. We identified a total of 37 *PRX* genes in guava, a number notably smaller than the 73 PRXs found in Arabidopsis, suggesting a unique pattern of gene family expansion. Our phylogenetic analysis revealed close relationships between *PRX* members of Arabidopsis and guava, with 12 *PgPRX* genes forming ortholog pairs with *AtPRXs*, indicating a specific expansion pattern. Furthermore, we explored the gene duplication events within the *PgPRX* gene family, distinguishing between tandem and segmental duplications. The significant prevalence of segmental duplications suggested the dominant role of this process in the expansion of the *PgPRX*s. Lastly, our study delved into the expression profiles of *PgPRX* genes across various guava tissues, highlighting significant variations in gene expression, underscoring the functional diversity of PgPRX proteins within the guava plant. Moreover, our investigation into the changes in peroxidase levels and gene expressions during postharvest storage of guava fruits provided valuable insights into maintaining fruit quality and extending shelf life. These findings contribute to a better understanding of the postharvest dynamics of peroxidase activity and regulatory genes, with potential applications in enhancing fruit quality during storage. Overall, our study serves as a foundational resource for future research on guava *PRX* genes and their roles in various aspects of plant growth and stress responses.

### Supplementary Information

Supplementary Information.

## Data Availability

The data presented in this study are contained within the article or supplementary material.
