# Supplementary material for: Comprehensive genomic exploration of class III peroxidase genes in guava unravels physiology, evolution, and postharvest storage responses
Source: Sci Rep. 2024 Jan 16;14:1446. doi: 10.1038/s41598-024-51961-4 (PMC10791677; doi:10.1038/s41598-024-51961-4)
Supplement: Supplementary file 1 — Supplementary Information. [file 41598_2024_51961_MOESM1_ESM.docx]

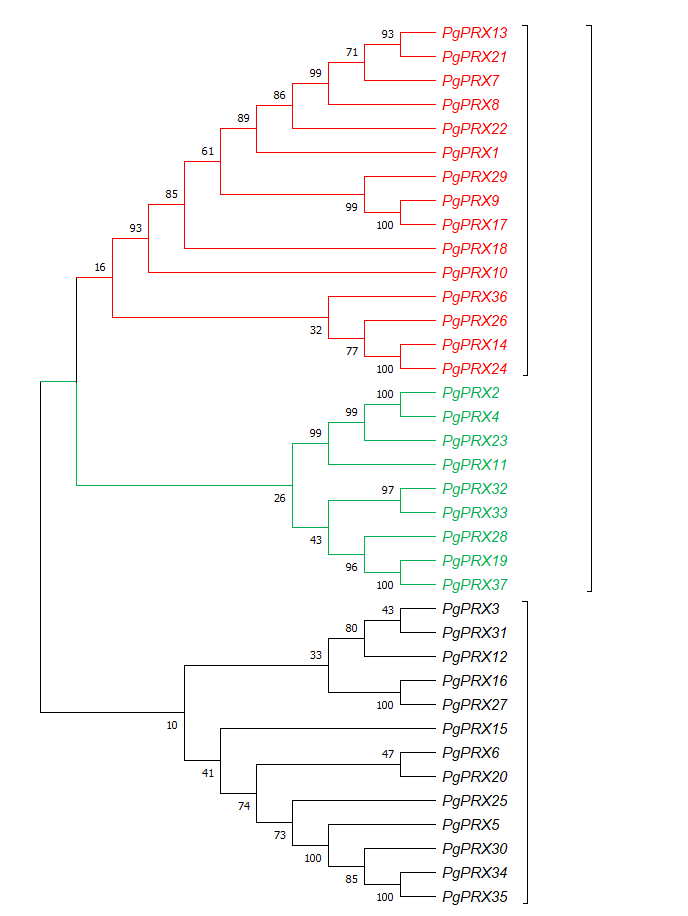


**Figure S1.** Phylogenetic Relationships between Class-III Peroxidase Genes in *Psidium guajava*. Distinct phylogenetic groups are highlighted with differently colored fonts.


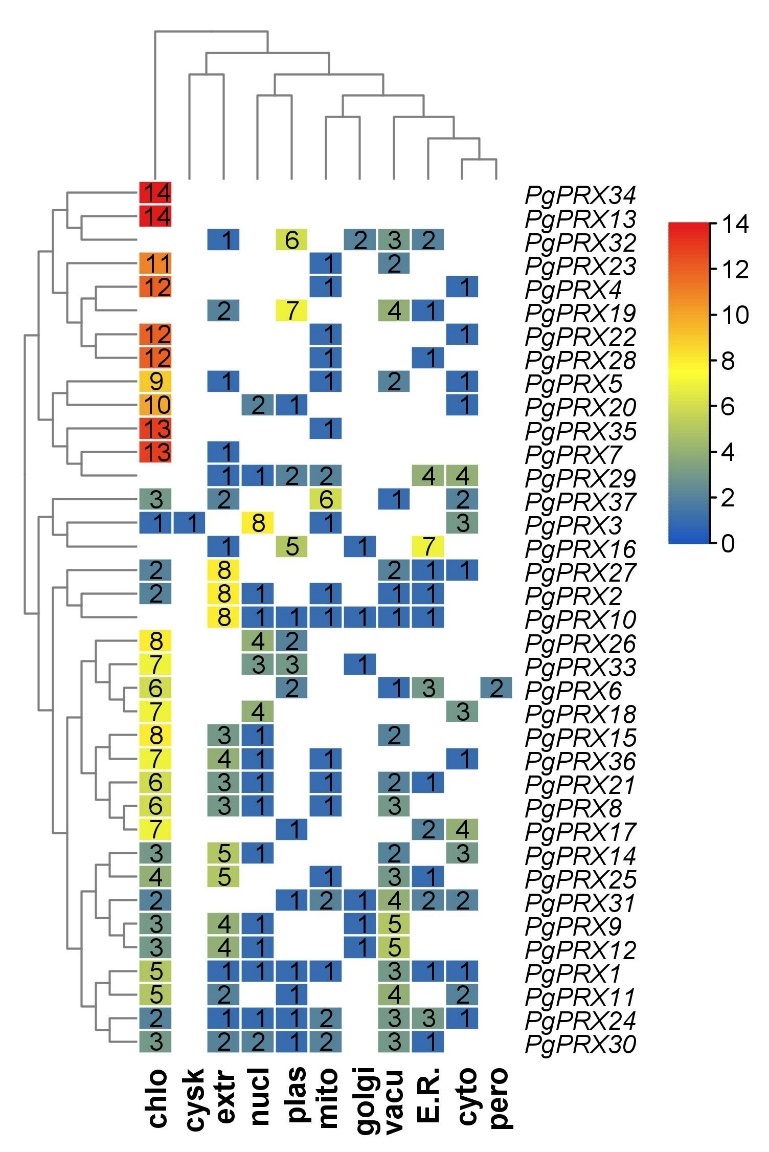


**Figure S2.** Predicting the subcellular localization of PgPRX Proteins. Signal intensity is depicted in a heatmap, with red indicating higher levels, blue representing lower levels, and white signifying no available data. Abbreviations: chlo – chloroplast; cysk – cytoplasmic skeleton; extr – extracellular region; nucl – nucleus; plas – plasma membrane; mito – mitochondria; golgi – Golgi apparatus; vacu – vacuole; E.R. – endoplasmic reticulum; cyto – cytoplasm; pero – peroxisomes.

**Table S1.** Details of Cis-regulatory elements detected in promoter regions of PgPRXs.

| **Gene Name** | **Start site** | **End site** | **Cis-element** | **Detail of cis-element** |
| --- | --- | --- | --- | --- |
| *PgPRX1* | 696 | 702 | ARE | cis-acting regulatory element essential for the anaerobic induction |
| *PgPRX1* | 996 | 1002 | ARE | cis-acting regulatory element essential for the anaerobic induction |
| *PgPRX1* | 731 | 737 | G-Box | cis-acting regulatory element involved in light responsiveness |
| *PgPRX1* | 832 | 837 | TGACG-motif | cis-acting regulatory element involved in the MeJA-responsiveness |
| *PgPRX1* | 832 | 837 | TGACG-motif | cis-acting regulatory element involved in the MeJA-responsiveness |
| *PgPRX1* | 44 | 49 | CAAT-box | common cis-acting element in promoter and enhancer regions |
| *PgPRX1* | 93 | 98 | CAAT-box | common cis-acting element in promoter and enhancer regions |
| *PgPRX1* | 191 | 196 | CAAT-box | common cis-acting element in promoter and enhancer regions |
| *PgPRX1* | 250 | 255 | CAAT-box | common cis-acting element in promoter and enhancer regions |
| *PgPRX1* | 332 | 337 | CAAT-box | common cis-acting element in promoter and enhancer regions |
| *PgPRX1* | 427 | 432 | CAAT-box | common cis-acting element in promoter and enhancer regions |
| *PgPRX1* | 475 | 480 | CAAT-box | common cis-acting element in promoter and enhancer regions |
| *PgPRX1* | 621 | 628 | P-box | gibberellin-responsive element |
| *PgPRX1* | 527 | 534 | GT1-motif | light responsive element |
| *PgPRX1* | 528 | 534 | GT1-motif | light responsive element |
| *PgPRX1* | 659 | 668 | Box 4 | part of a conserved DNA module involved in light responsiveness |
| *PgPRX1* | 152 | 158 | Box 4 | part of a conserved DNA module involved in light responsiveness |
| *PgPRX1* | 205 | 211 | Box 4 | part of a conserved DNA module involved in light responsiveness |
| *PgPRX1* | 412 | 418 | Box 4 | part of a conserved DNA module involved in light responsiveness |
| *PgPRX1* | 471 | 477 | Box 4 | part of a conserved DNA module involved in light responsiveness |
| *PgPRX1* | 726 | 732 | TCT-motif | part of a light responsive element |
| *PgPRX2* | 616 | 625 | TC-rich repeats | cis-acting element involved in defense and stress responsiveness |
| *PgPRX2* | 165 | 171 | LTR | cis-acting element involved in low-temperature responsiveness |
| *PgPRX2* | 325 | 331 | LTR | cis-acting element involved in low-temperature responsiveness |
| *PgPRX2* | 708 | 713 | ABRE | cis-acting element involved in the abscisic acid responsiveness |
| *PgPRX2* | 826 | 831 | ABRE | cis-acting element involved in the abscisic acid responsiveness |
| *PgPRX2* | 20 | 26 | ARE | cis-acting regulatory element essential for the anaerobic induction |
| *PgPRX2* | 437 | 443 | ARE | cis-acting regulatory element essential for the anaerobic induction |
| *PgPRX2* | 707 | 713 | G-Box | cis-acting regulatory element involved in light responsiveness |
| *PgPRX2* | 826 | 832 | G-Box | cis-acting regulatory element involved in light responsiveness |
| *PgPRX2* | 957 | 966 | O2-site | cis-acting regulatory element involved in zein metabolism regulation |
| *PgPRX2* | 94 | 99 | CAAT-box | common cis-acting element in promoter and enhancer regions |
| *PgPRX2* | 192 | 197 | CAAT-box | common cis-acting element in promoter and enhancer regions |
| *PgPRX2* | 306 | 311 | CAAT-box | common cis-acting element in promoter and enhancer regions |
| *PgPRX2* | 424 | 429 | CAAT-box | common cis-acting element in promoter and enhancer regions |
| *PgPRX2* | 518 | 523 | CAAT-box | common cis-acting element in promoter and enhancer regions |
| *PgPRX2* | 571 | 576 | CAAT-box | common cis-acting element in promoter and enhancer regions |
| *PgPRX2* | 574 | 579 | CAAT-box | common cis-acting element in promoter and enhancer regions |
| *PgPRX2* | 588 | 593 | CAAT-box | common cis-acting element in promoter and enhancer regions |
| *PgPRX2* | 684 | 689 | CAAT-box | common cis-acting element in promoter and enhancer regions |
| *PgPRX2* | 860 | 867 | P-box | gibberellin-responsive element |
| *PgPRX2* | 511 | 517 | Box 4 | part of a conserved DNA module involved in light responsiveness |
| *PgPRX3* | 732 | 738 | A-box | cis-acting regulatory element |
| *PgPRX3* | 167 | 173 | ARE | cis-acting regulatory element essential for the anaerobic induction |
| *PgPRX3* | 67 | 72 | CAAT-box | common cis-acting element in promoter and enhancer regions |
| *PgPRX3* | 196 | 201 | CAAT-box | common cis-acting element in promoter and enhancer regions |
| *PgPRX3* | 339 | 344 | CAAT-box | common cis-acting element in promoter and enhancer regions |
| *PgPRX3* | 431 | 436 | CAAT-box | common cis-acting element in promoter and enhancer regions |
| *PgPRX3* | 493 | 498 | CAAT-box | common cis-acting element in promoter and enhancer regions |
| *PgPRX3* | 500 | 505 | CAAT-box | common cis-acting element in promoter and enhancer regions |
| *PgPRX3* | 518 | 523 | CAAT-box | common cis-acting element in promoter and enhancer regions |
| *PgPRX3* | 741 | 746 | CAAT-box | common cis-acting element in promoter and enhancer regions |
| *PgPRX3* | 624 | 630 | GC-motif | enhancer-like element involved in anoxic specific inducibility |
| *PgPRX3* | 679 | 685 | GC-motif | enhancer-like element involved in anoxic specific inducibility |
| *PgPRX3* | 114 | 121 | P-box | gibberellin-responsive element |
| *PgPRX3* | 561 | 571 | GT1-motif | light responsive element |
| *PgPRX3* | 294 | 300 | Box 4 | part of a conserved DNA module involved in light responsiveness |
| *PgPRX3* | 496 | 502 | Box 4 | part of a conserved DNA module involved in light responsiveness |
| *PgPRX3* | 395 | 404 | Box 4 | part of a conserved DNA module involved in light responsiveness |
| *PgPRX3* | 271 | 277 | TCT-motif | part of a light responsive element |
| *PgPRX3* | 446 | 454 | TCT-motif | part of a light responsive element |
| *PgPRX3* | 67 | 76.5 | TCT-motif | part of a light responsive element |
| *PgPRX4* | 100 | 109 | ABRE | cis-acting element involved in the abscisic acid responsiveness |
| *PgPRX4* | 102 | 107 | ABRE | cis-acting element involved in the abscisic acid responsiveness |
| *PgPRX4* | 765 | 770 | ABRE | cis-acting element involved in the abscisic acid responsiveness |
| *PgPRX4* | 795 | 800 | ABRE | cis-acting element involved in the abscisic acid responsiveness |
| *PgPRX4* | 764 | 770 | G-Box | cis-acting regulatory element involved in light responsiveness |
| *PgPRX4* | 795 | 801 | G-Box | cis-acting regulatory element involved in light responsiveness |
| *PgPRX4* | 102 | 108 | G-Box | cis-acting regulatory element involved in light responsiveness |
| *PgPRX4* | 704 | 710 | G-Box | cis-acting regulatory element involved in light responsiveness |
| *PgPRX4* | 944 | 949 | TGACG-motif | cis-acting regulatory element involved in the MeJA-responsiveness |
| *PgPRX4* | 944 | 949 | TGACG-motif | cis-acting regulatory element involved in the MeJA-responsiveness |
| *PgPRX4* | 28 | 33 | CAAT-box | common cis-acting element in promoter and enhancer regions |
| *PgPRX4* | 68 | 73 | CAAT-box | common cis-acting element in promoter and enhancer regions |
| *PgPRX4* | 150 | 155 | CAAT-box | common cis-acting element in promoter and enhancer regions |
| *PgPRX4* | 209 | 214 | CAAT-box | common cis-acting element in promoter and enhancer regions |
| *PgPRX4* | 258 | 263 | CAAT-box | common cis-acting element in promoter and enhancer regions |
| *PgPRX4* | 312 | 317 | CAAT-box | common cis-acting element in promoter and enhancer regions |
| *PgPRX4* | 384 | 389 | CAAT-box | common cis-acting element in promoter and enhancer regions |
| *PgPRX4* | 680 | 685 | CAAT-box | common cis-acting element in promoter and enhancer regions |
| *PgPRX4* | 759 | 764 | CAAT-box | common cis-acting element in promoter and enhancer regions |
| *PgPRX4* | 883 | 888 | CAAT-box | common cis-acting element in promoter and enhancer regions |
| *PgPRX4* | 461 | 468 | P-box | gibberellin-responsive element |
| *PgPRX4* | 829 | 836 | P-box | gibberellin-responsive element |
| *PgPRX4* | 243 | 249 | Box 4 | part of a conserved DNA module involved in light responsiveness |
| *PgPRX4* | 637 | 643 | Box 4 | part of a conserved DNA module involved in light responsiveness |
| *PgPRX4* | 227 | 233 | TCT-motif | part of a light responsive element |
| *PgPRX4* | 610 | 623 | AT1-motif | part of a light responsive module |
| *PgPRX5* | 8 | 14 | TGA-element | auxin-responsive element |
| *PgPRX5* | 722 | 727 | ABRE | cis-acting element involved in the abscisic acid responsiveness |
| *PgPRX5* | 442 | 448 | ARE | cis-acting regulatory element essential for the anaerobic induction |
| *PgPRX5* | 883 | 889 | G-Box | cis-acting regulatory element involved in light responsiveness |
| *PgPRX5* | 721 | 727 | G-Box | cis-acting regulatory element involved in light responsiveness |
| *PgPRX5* | 44 | 49 | CAAT-box | common cis-acting element in promoter and enhancer regions |
| *PgPRX5* | 618 | 623 | CAAT-box | common cis-acting element in promoter and enhancer regions |
| *PgPRX5* | 966 | 971 | CAAT-box | common cis-acting element in promoter and enhancer regions |
| *PgPRX5* | 273 | 280 | GT1-motif | light responsive element |
| *PgPRX5* | 274 | 280 | GT1-motif | light responsive element |
| *PgPRX5* | 420 | 430 | TCT-motif | part of a light responsive element |
| *PgPRX5* | 487 | 495 | TCT-motif | part of a light responsive element |
| *PgPRX6* | 933 | 939 | TGA-element | auxin-responsive element |
| *PgPRX6* | 186 | 195 | ABRE | cis-acting element involved in the abscisic acid responsiveness |
| *PgPRX6* | 321 | 327 | ABRE | cis-acting element involved in the abscisic acid responsiveness |
| *PgPRX6* | 322 | 327 | ABRE | cis-acting element involved in the abscisic acid responsiveness |
| *PgPRX6* | 777 | 782 | ABRE | cis-acting element involved in the abscisic acid responsiveness |
| *PgPRX6* | 747 | 753 | ARE | cis-acting regulatory element essential for the anaerobic induction |
| *PgPRX6* | 318 | 327 | G-Box | cis-acting regulatory element involved in light responsiveness |
| *PgPRX6* | 321 | 327 | G-Box | cis-acting regulatory element involved in light responsiveness |
| *PgPRX6* | 776 | 782 | G-Box | cis-acting regulatory element involved in light responsiveness |
| *PgPRX6* | 168 | 174 | G-Box | cis-acting regulatory element involved in light responsiveness |
| *PgPRX6* | 321 | 327 | G-Box | cis-acting regulatory element involved in light responsiveness |
| *PgPRX6* | 2 | 9 | GCN4_motif | cis-regulatory element involved in endosperm expression |
| *PgPRX6* | 593 | 600 | GCN4_motif | cis-regulatory element involved in endosperm expression |
| *PgPRX6* | 83 | 88 | CAAT-box | common cis-acting element in promoter and enhancer regions |
| *PgPRX6* | 119 | 124 | CAAT-box | common cis-acting element in promoter and enhancer regions |
| *PgPRX6* | 127 | 132 | CAAT-box | common cis-acting element in promoter and enhancer regions |
| *PgPRX6* | 490 | 495 | CAAT-box | common cis-acting element in promoter and enhancer regions |
| *PgPRX6* | 920 | 925 | CAAT-box | common cis-acting element in promoter and enhancer regions |
| *PgPRX6* | 282 | 290 | Box 4 | part of a conserved DNA module involved in light responsiveness |
| *PgPRX6* | 465 | 473 | TCT-motif | part of a light responsive element |
| *PgPRX6* | 496 | 504 | TCT-motif | part of a light responsive element |
| *PgPRX7* | 827 | 836 | TC-rich repeats | cis-acting element involved in defense and stress responsiveness |
| *PgPRX7* | 592 | 599 | ABRE | cis-acting element involved in the abscisic acid responsiveness |
| *PgPRX7* | 604 | 609 | ABRE | cis-acting element involved in the abscisic acid responsiveness |
| *PgPRX7* | 639 | 644 | ABRE | cis-acting element involved in the abscisic acid responsiveness |
| *PgPRX7* | 761 | 766 | ABRE | cis-acting element involved in the abscisic acid responsiveness |
| *PgPRX7* | 835 | 840 | ABRE | cis-acting element involved in the abscisic acid responsiveness |
| *PgPRX7* | 64 | 70 | ARE | cis-acting regulatory element essential for the anaerobic induction |
| *PgPRX7* | 692 | 698 | ARE | cis-acting regulatory element essential for the anaerobic induction |
| *PgPRX7* | 834 | 840 | G-Box | cis-acting regulatory element involved in light responsiveness |
| *PgPRX7* | 248 | 254 | G-Box | cis-acting regulatory element involved in light responsiveness |
| *PgPRX7* | 604 | 610 | G-Box | cis-acting regulatory element involved in light responsiveness |
| *PgPRX7* | 638 | 644 | G-Box | cis-acting regulatory element involved in light responsiveness |
| *PgPRX7* | 761 | 767 | G-Box | cis-acting regulatory element involved in light responsiveness |
| *PgPRX7* | 610 | 618 | RY-element | cis-acting regulatory element involved in seed-specific regulation |
| *PgPRX7* | 562 | 567 | TGACG-motif | cis-acting regulatory element involved in the MeJA-responsiveness |
| *PgPRX7* | 812 | 817 | TGACG-motif | cis-acting regulatory element involved in the MeJA-responsiveness |
| *PgPRX7* | 562 | 567 | TGACG-motif | cis-acting regulatory element involved in the MeJA-responsiveness |
| *PgPRX7* | 812 | 817 | TGACG-motif | cis-acting regulatory element involved in the MeJA-responsiveness |
| *PgPRX7* | 47 | 52 | CAAT-box | common cis-acting element in promoter and enhancer regions |
| *PgPRX7* | 78 | 83 | CAAT-box | common cis-acting element in promoter and enhancer regions |
| *PgPRX7* | 85 | 90 | CAAT-box | common cis-acting element in promoter and enhancer regions |
| *PgPRX7* | 224 | 229 | CAAT-box | common cis-acting element in promoter and enhancer regions |
| *PgPRX7* | 620 | 625 | CAAT-box | common cis-acting element in promoter and enhancer regions |
| *PgPRX7* | 711 | 716 | CAAT-box | common cis-acting element in promoter and enhancer regions |
| *PgPRX7* | 785 | 790 | CAAT-box | common cis-acting element in promoter and enhancer regions |
| *PgPRX7* | 857 | 862 | CAAT-box | common cis-acting element in promoter and enhancer regions |
| *PgPRX7* | 980 | 986 | GC-motif | enhancer-like element involved in anoxic specific inducibility |
| *PgPRX7* | 625 | 632 | P-box | gibberellin-responsive element |
| *PgPRX7* | 738 | 748 | TCT-motif | part of a light responsive element |
| *PgPRX7* | 551 | 558 | TCT-motif | part of a light responsive element |
| *PgPRX7* | 957 | 963 | TCT-motif | part of a light responsive element |
| *PgPRX8* | 587 | 596 | TC-rich repeats | cis-acting element involved in defense and stress responsiveness |
| *PgPRX8* | 212 | 222 | TCA-element | cis-acting element involved in salicylic acid responsiveness |
| *PgPRX8* | 352 | 361 | TCA-element | cis-acting element involved in salicylic acid responsiveness |
| *PgPRX8* | 118 | 124 | ARE | cis-acting regulatory element essential for the anaerobic induction |
| *PgPRX8* | 908 | 914 | G-Box | cis-acting regulatory element involved in light responsiveness |
| *PgPRX8* | 974 | 979 | TGACG-motif | cis-acting regulatory element involved in the MeJA-responsiveness |
| *PgPRX8* | 974 | 979 | TGACG-motif | cis-acting regulatory element involved in the MeJA-responsiveness |
| *PgPRX8* | 327 | 333 | CAT-box | cis-acting regulatory element related to meristem expression |
| *PgPRX8* | 671 | 676 | CAAT-box | common cis-acting element in promoter and enhancer regions |
| *PgPRX8* | 799 | 804 | CAAT-box | common cis-acting element in promoter and enhancer regions |
| *PgPRX8* | 249 | 256 | P-box | gibberellin-responsive element |
| *PgPRX8* | 281 | 289 | AE-box | part of a module for light response |
| *PgPRX9* | 336 | 342 | TGA-element | auxin-responsive element |
| *PgPRX9* | 958 | 964 | LTR | cis-acting element involved in low-temperature responsiveness |
| *PgPRX9* | 109 | 118 | TCA-element | cis-acting element involved in salicylic acid responsiveness |
| *PgPRX9* | 861 | 870 | TCA-element | cis-acting element involved in salicylic acid responsiveness |
| *PgPRX9* | 763 | 772 | ABRE | cis-acting element involved in the abscisic acid responsiveness |
| *PgPRX9* | 765 | 770 | ABRE | cis-acting element involved in the abscisic acid responsiveness |
| *PgPRX9* | 765 | 771 | G-Box | cis-acting regulatory element involved in light responsiveness |
| *PgPRX9* | 70 | 75 | TGACG-motif | cis-acting regulatory element involved in the MeJA-responsiveness |
| *PgPRX9* | 119 | 124 | TGACG-motif | cis-acting regulatory element involved in the MeJA-responsiveness |
| *PgPRX9* | 745 | 750 | TGACG-motif | cis-acting regulatory element involved in the MeJA-responsiveness |
| *PgPRX9* | 70 | 75 | TGACG-motif | cis-acting regulatory element involved in the MeJA-responsiveness |
| *PgPRX9* | 119 | 124 | TGACG-motif | cis-acting regulatory element involved in the MeJA-responsiveness |
| *PgPRX9* | 745 | 750 | TGACG-motif | cis-acting regulatory element involved in the MeJA-responsiveness |
| *PgPRX9* | 537 | 542 | CAAT-box | common cis-acting element in promoter and enhancer regions |
| *PgPRX9* | 787 | 792 | CAAT-box | common cis-acting element in promoter and enhancer regions |
| *PgPRX9* | 809 | 816 | CAAT-box | common cis-acting element in promoter and enhancer regions |
| *PgPRX9* | 852 | 862 | CAAT-box | common cis-acting element in promoter and enhancer regions |
| *PgPRX9* | 397 | 405 | HD-Zip 1 | element involved in differentiation of the palisade mesophyll cells |
| *PgPRX9* | 426 | 433 | P-box | gibberellin-responsive element |
| *PgPRX9* | 806 | 813 | P-box | gibberellin-responsive element |
| *PgPRX9* | 577 | 583 | GT1-motif | light responsive element |
| *PgPRX9* | 198 | 206 | TCT-motif | part of a light responsive element |
| *PgPRX9* | 203 | 210 | TCT-motif | part of a light responsive element |
| *PgPRX9* | 619 | 628 | TCT-motif | part of a light responsive element |
| *PgPRX9* | 74 | 80 | TCT-motif | part of a light responsive element |
| *PgPRX9* | 70 | 78 | AuxRE | part of an auxin-responsive element |
| *PgPRX9* | 116 | 124 | AuxRE | part of an auxin-responsive element |
| *PgPRX10* | 675 | 681 | ABRE | cis-acting element involved in the abscisic acid responsiveness |
| *PgPRX10* | 676 | 681 | ABRE | cis-acting element involved in the abscisic acid responsiveness |
| *PgPRX10* | 675 | 681 | G-Box | cis-acting regulatory element involved in light responsiveness |
| *PgPRX10* | 675 | 681 | G-Box | cis-acting regulatory element involved in light responsiveness |
| *PgPRX10* | 852 | 857 | TGACG-motif | cis-acting regulatory element involved in the MeJA-responsiveness |
| *PgPRX10* | 979 | 984 | TGACG-motif | cis-acting regulatory element involved in the MeJA-responsiveness |
| *PgPRX10* | 852 | 857 | TGACG-motif | cis-acting regulatory element involved in the MeJA-responsiveness |
| *PgPRX10* | 979 | 984 | TGACG-motif | cis-acting regulatory element involved in the MeJA-responsiveness |
| *PgPRX10* | 67 | 76 | O2-site | cis-acting regulatory element involved in zein metabolism regulation |
| *PgPRX10* | 758 | 764 | CAT-box | cis-acting regulatory element related to meristem expression |
| *PgPRX10* | 834 | 840 | CAT-box | cis-acting regulatory element related to meristem expression |
| *PgPRX10* | 951 | 957 | CAT-box | cis-acting regulatory element related to meristem expression |
| *PgPRX10* | 84 | 89 | CAAT-box | common cis-acting element in promoter and enhancer regions |
| *PgPRX10* | 97 | 102 | CAAT-box | common cis-acting element in promoter and enhancer regions |
| *PgPRX10* | 301 | 306 | CAAT-box | common cis-acting element in promoter and enhancer regions |
| *PgPRX10* | 754 | 759 | CAAT-box | common cis-acting element in promoter and enhancer regions |
| *PgPRX10* | 867 | 872 | CAAT-box | common cis-acting element in promoter and enhancer regions |
| *PgPRX11* | 581 | 587 | TGA-element | auxin-responsive element |
| *PgPRX11* | 678 | 684 | LTR | cis-acting element involved in low-temperature responsiveness |
| *PgPRX11* | 188 | 193 | ABRE | cis-acting element involved in the abscisic acid responsiveness |
| *PgPRX11* | 272 | 277 | ABRE | cis-acting element involved in the abscisic acid responsiveness |
| *PgPRX11* | 904 | 910 | ARE | cis-acting regulatory element essential for the anaerobic induction |
| *PgPRX11* | 187 | 193 | G-Box | cis-acting regulatory element involved in light responsiveness |
| *PgPRX11* | 243 | 249 | G-Box | cis-acting regulatory element involved in light responsiveness |
| *PgPRX11* | 271 | 277 | G-Box | cis-acting regulatory element involved in light responsiveness |
| *PgPRX11* | 186 | 191 | TGACG-motif | cis-acting regulatory element involved in the MeJA-responsiveness |
| *PgPRX11* | 186 | 191 | TGACG-motif | cis-acting regulatory element involved in the MeJA-responsiveness |
| *PgPRX11* | 61 | 66 | CAAT-box | common cis-acting element in promoter and enhancer regions |
| *PgPRX11* | 85 | 90 | CAAT-box | common cis-acting element in promoter and enhancer regions |
| *PgPRX11* | 297 | 302 | CAAT-box | common cis-acting element in promoter and enhancer regions |
| *PgPRX11* | 331 | 336 | CAAT-box | common cis-acting element in promoter and enhancer regions |
| *PgPRX11* | 463 | 468 | CAAT-box | common cis-acting element in promoter and enhancer regions |
| *PgPRX11* | 552 | 557 | CAAT-box | common cis-acting element in promoter and enhancer regions |
| *PgPRX11* | 607 | 612 | CAAT-box | common cis-acting element in promoter and enhancer regions |
| *PgPRX11* | 824 | 829 | CAAT-box | common cis-acting element in promoter and enhancer regions |
| *PgPRX11* | 553 | 561.5 | HD-Zip 1 | element involved in differentiation of the palisade mesophyll cells |
| *PgPRX12* | 576 | 585 | TC-rich repeats | cis-acting element involved in defense and stress responsiveness |
| *PgPRX12* | 448 | 453 | TGACG-motif | cis-acting regulatory element involved in the MeJA-responsiveness |
| *PgPRX12* | 575 | 580 | TGACG-motif | cis-acting regulatory element involved in the MeJA-responsiveness |
| *PgPRX12* | 650 | 655 | TGACG-motif | cis-acting regulatory element involved in the MeJA-responsiveness |
| *PgPRX12* | 931 | 936 | TGACG-motif | cis-acting regulatory element involved in the MeJA-responsiveness |
| *PgPRX12* | 448 | 453 | TGACG-motif | cis-acting regulatory element involved in the MeJA-responsiveness |
| *PgPRX12* | 575 | 580 | TGACG-motif | cis-acting regulatory element involved in the MeJA-responsiveness |
| *PgPRX12* | 650 | 655 | TGACG-motif | cis-acting regulatory element involved in the MeJA-responsiveness |
| *PgPRX12* | 931 | 936 | TGACG-motif | cis-acting regulatory element involved in the MeJA-responsiveness |
| *PgPRX12* | 552 | 559 | GCN4_motif | cis-regulatory element involved in endosperm expression |
| *PgPRX12* | 86 | 91 | CAAT-box | common cis-acting element in promoter and enhancer regions |
| *PgPRX12* | 617 | 622 | CAAT-box | common cis-acting element in promoter and enhancer regions |
| *PgPRX12* | 754 | 759 | CAAT-box | common cis-acting element in promoter and enhancer regions |
| *PgPRX12* | 834 | 841 | CAAT-box | common cis-acting element in promoter and enhancer regions |
| *PgPRX12* | 900 | 905 | CAAT-box | common cis-acting element in promoter and enhancer regions |
| *PgPRX12* | 20 | 27 | P-box | gibberellin-responsive element |
| *PgPRX12* | 664 | 670 | GT1-motif | light responsive element |
| *PgPRX12* | 584 | 590 | Box 4 | part of a conserved DNA module involved in light responsiveness |
| *PgPRX12* | 761 | 770 | TCT-motif | part of a light responsive element |
| *PgPRX13* | 179 | 185 | TGA-element | auxin-responsive element |
| *PgPRX13* | 577 | 586 | TC-rich repeats | cis-acting element involved in defense and stress responsiveness |
| *PgPRX13* | 859 | 868 | TCA-element | cis-acting element involved in salicylic acid responsiveness |
| *PgPRX13* | 782 | 787 | ABRE | cis-acting element involved in the abscisic acid responsiveness |
| *PgPRX13* | 876 | 881 | ABRE | cis-acting element involved in the abscisic acid responsiveness |
| *PgPRX13* | 782 | 788 | G-Box | cis-acting regulatory element involved in light responsiveness |
| *PgPRX13* | 874 | 884 | G-Box | cis-acting regulatory element involved in light responsiveness |
| *PgPRX13* | 875 | 881 | G-Box | cis-acting regulatory element involved in light responsiveness |
| *PgPRX13* | 151 | 156 | CAAT-box | common cis-acting element in promoter and enhancer regions |
| *PgPRX13* | 250 | 255 | CAAT-box | common cis-acting element in promoter and enhancer regions |
| *PgPRX13* | 270 | 275 | CAAT-box | common cis-acting element in promoter and enhancer regions |
| *PgPRX13* | 280 | 285 | CAAT-box | common cis-acting element in promoter and enhancer regions |
| *PgPRX13* | 320 | 325 | CAAT-box | common cis-acting element in promoter and enhancer regions |
| *PgPRX13* | 462 | 467 | CAAT-box | common cis-acting element in promoter and enhancer regions |
| *PgPRX13* | 468 | 473 | CAAT-box | common cis-acting element in promoter and enhancer regions |
| *PgPRX13* | 587 | 592 | CAAT-box | common cis-acting element in promoter and enhancer regions |
| *PgPRX13* | 986 | 991 | CAAT-box | common cis-acting element in promoter and enhancer regions |
| *PgPRX13* | 170 | 176 | GC-motif | enhancer-like element involved in anoxic specific inducibility |
| *PgPRX13* | 721 | 730 | Box 4 | part of a conserved DNA module involved in light responsiveness |
| *PgPRX13* | 873 | 882 | TCT-motif | part of a light responsive element |
| *PgPRX13* | 599 | 606 | TCT-motif | part of a light responsive element |
| *PgPRX14* | 362 | 371 | TCA-element | cis-acting element involved in salicylic acid responsiveness |
| *PgPRX14* | 105 | 111 | ABRE | cis-acting element involved in the abscisic acid responsiveness |
| *PgPRX14* | 106 | 111 | ABRE | cis-acting element involved in the abscisic acid responsiveness |
| *PgPRX14* | 279 | 284 | ABRE | cis-acting element involved in the abscisic acid responsiveness |
| *PgPRX14* | 410 | 417 | ABRE | cis-acting element involved in the abscisic acid responsiveness |
| *PgPRX14* | 105 | 111 | G-Box | cis-acting regulatory element involved in light responsiveness |
| *PgPRX14* | 105 | 111 | G-Box | cis-acting regulatory element involved in light responsiveness |
| *PgPRX14* | 279 | 285 | G-Box | cis-acting regulatory element involved in light responsiveness |
| *PgPRX14* | 685 | 691 | G-Box | cis-acting regulatory element involved in light responsiveness |
| *PgPRX14* | 281 | 286 | TGACG-motif | cis-acting regulatory element involved in the MeJA-responsiveness |
| *PgPRX14* | 691 | 696 | TGACG-motif | cis-acting regulatory element involved in the MeJA-responsiveness |
| *PgPRX14* | 812 | 817 | TGACG-motif | cis-acting regulatory element involved in the MeJA-responsiveness |
| *PgPRX14* | 281 | 286 | TGACG-motif | cis-acting regulatory element involved in the MeJA-responsiveness |
| *PgPRX14* | 691 | 696 | TGACG-motif | cis-acting regulatory element involved in the MeJA-responsiveness |
| *PgPRX14* | 812 | 817 | TGACG-motif | cis-acting regulatory element involved in the MeJA-responsiveness |
| *PgPRX14* | 278 | 288 | O2-site | cis-acting regulatory element involved in zein metabolism regulation |
| *PgPRX14* | 725 | 730 | CAAT-box | common cis-acting element in promoter and enhancer regions |
| *PgPRX14* | 794 | 799 | CAAT-box | common cis-acting element in promoter and enhancer regions |
| *PgPRX14* | 344 | 353 | Box 4 | part of a conserved DNA module involved in light responsiveness |
| *PgPRX14* | 351 | 357 | Box 4 | part of a conserved DNA module involved in light responsiveness |
| *PgPRX14* | 880 | 886 | Box 4 | part of a conserved DNA module involved in light responsiveness |
| *PgPRX14* | 58 | 65 | TCT-motif | part of a light responsive element |
| *PgPRX15* | 407 | 414 | TATC-box | cis-acting element involved in gibberellin-responsiveness |
| *PgPRX15* | 210 | 215 | ABRE | cis-acting element involved in the abscisic acid responsiveness |
| *PgPRX15* | 423 | 428 | ABRE | cis-acting element involved in the abscisic acid responsiveness |
| *PgPRX15* | 651 | 656 | ABRE | cis-acting element involved in the abscisic acid responsiveness |
| *PgPRX15* | 842 | 847 | ABRE | cis-acting element involved in the abscisic acid responsiveness |
| *PgPRX15* | 210 | 216 | G-Box | cis-acting regulatory element involved in light responsiveness |
| *PgPRX15* | 651 | 657 | G-Box | cis-acting regulatory element involved in light responsiveness |
| *PgPRX15* | 210 | 218 | G-Box | cis-acting regulatory element involved in light responsiveness |
| *PgPRX15* | 423 | 429 | G-Box | cis-acting regulatory element involved in light responsiveness |
| *PgPRX15* | 842 | 848 | G-Box | cis-acting regulatory element involved in light responsiveness |
| *PgPRX15* | 839 | 844 | TGACG-motif | cis-acting regulatory element involved in the MeJA-responsiveness |
| *PgPRX15* | 839 | 844 | TGACG-motif | cis-acting regulatory element involved in the MeJA-responsiveness |
| *PgPRX15* | 20 | 25 | CAAT-box | common cis-acting element in promoter and enhancer regions |
| *PgPRX15* | 109 | 114 | CAAT-box | common cis-acting element in promoter and enhancer regions |
| *PgPRX15* | 168 | 173 | CAAT-box | common cis-acting element in promoter and enhancer regions |
| *PgPRX15* | 242 | 247 | CAAT-box | common cis-acting element in promoter and enhancer regions |
| *PgPRX15* | 267 | 272 | CAAT-box | common cis-acting element in promoter and enhancer regions |
| *PgPRX15* | 297 | 302 | CAAT-box | common cis-acting element in promoter and enhancer regions |
| *PgPRX15* | 357 | 362 | CAAT-box | common cis-acting element in promoter and enhancer regions |
| *PgPRX15* | 415 | 420 | CAAT-box | common cis-acting element in promoter and enhancer regions |
| *PgPRX15* | 534 | 539 | CAAT-box | common cis-acting element in promoter and enhancer regions |
| *PgPRX15* | 555 | 560 | CAAT-box | common cis-acting element in promoter and enhancer regions |
| *PgPRX15* | 575 | 580 | CAAT-box | common cis-acting element in promoter and enhancer regions |
| *PgPRX15* | 622 | 627 | CAAT-box | common cis-acting element in promoter and enhancer regions |
| *PgPRX15* | 628 | 633 | CAAT-box | common cis-acting element in promoter and enhancer regions |
| *PgPRX15* | 673 | 678 | CAAT-box | common cis-acting element in promoter and enhancer regions |
| *PgPRX15* | 286 | 293 | P-box | gibberellin-responsive element |
| *PgPRX15* | 825 | 831 | GT1-motif | light responsive element |
| *PgPRX15* | 312 | 318 | Box 4 | part of a conserved DNA module involved in light responsiveness |
| *PgPRX15* | 519 | 527 | TCT-motif | part of a light responsive element |
| *PgPRX15* | 886 | 892 | TCT-motif | part of a light responsive element |
| *PgPRX16* | 430 | 437 | TATC-box | cis-acting element involved in gibberellin-responsiveness |
| *PgPRX16* | 269 | 274 | ABRE | cis-acting element involved in the abscisic acid responsiveness |
| *PgPRX16* | 313 | 318 | ABRE | cis-acting element involved in the abscisic acid responsiveness |
| *PgPRX16* | 768 | 773 | ABRE | cis-acting element involved in the abscisic acid responsiveness |
| *PgPRX16* | 806 | 811 | ABRE | cis-acting element involved in the abscisic acid responsiveness |
| *PgPRX16* | 850 | 855 | ABRE | cis-acting element involved in the abscisic acid responsiveness |
| *PgPRX16* | 268 | 276 | G-Box | cis-acting regulatory element involved in light responsiveness |
| *PgPRX16* | 269 | 275 | G-Box | cis-acting regulatory element involved in light responsiveness |
| *PgPRX16* | 313 | 319 | G-Box | cis-acting regulatory element involved in light responsiveness |
| *PgPRX16* | 768 | 774 | G-Box | cis-acting regulatory element involved in light responsiveness |
| *PgPRX16* | 862 | 868 | G-Box | cis-acting regulatory element involved in light responsiveness |
| *PgPRX16* | 806 | 812 | G-Box | cis-acting regulatory element involved in light responsiveness |
| *PgPRX16* | 850 | 856 | G-Box | cis-acting regulatory element involved in light responsiveness |
| *PgPRX16* | 56 | 61 | TGACG-motif | cis-acting regulatory element involved in the MeJA-responsiveness |
| *PgPRX16* | 56 | 61 | TGACG-motif | cis-acting regulatory element involved in the MeJA-responsiveness |
| *PgPRX16* | 182 | 188 | CAT-box | cis-acting regulatory element related to meristem expression |
| *PgPRX16* | 126 | 131 | CAAT-box | common cis-acting element in promoter and enhancer regions |
| *PgPRX16* | 163 | 168 | CAAT-box | common cis-acting element in promoter and enhancer regions |
| *PgPRX16* | 255 | 260 | CAAT-box | common cis-acting element in promoter and enhancer regions |
| *PgPRX16* | 383 | 388 | CAAT-box | common cis-acting element in promoter and enhancer regions |
| *PgPRX16* | 434 | 439 | CAAT-box | common cis-acting element in promoter and enhancer regions |
| *PgPRX16* | 512 | 517 | CAAT-box | common cis-acting element in promoter and enhancer regions |
| *PgPRX16* | 623 | 628 | CAAT-box | common cis-acting element in promoter and enhancer regions |
| *PgPRX16* | 699 | 704 | CAAT-box | common cis-acting element in promoter and enhancer regions |
| *PgPRX16* | 705 | 710 | CAAT-box | common cis-acting element in promoter and enhancer regions |
| *PgPRX16* | 726 | 731 | CAAT-box | common cis-acting element in promoter and enhancer regions |
| *PgPRX16* | 895 | 900 | CAAT-box | common cis-acting element in promoter and enhancer regions |
| *PgPRX16* | 959 | 964 | CAAT-box | common cis-acting element in promoter and enhancer regions |
| *PgPRX16* | 620 | 629 | Box 4 | part of a conserved DNA module involved in light responsiveness |
| *PgPRX16* | 316 | 322 | TCT-motif | part of a light responsive element |
| *PgPRX17* | 318 | 327 | ACE | cis-acting element involved in light responsiveness |
| *PgPRX17* | 739 | 744 | ABRE | cis-acting element involved in the abscisic acid responsiveness |
| *PgPRX17* | 791 | 796 | ABRE | cis-acting element involved in the abscisic acid responsiveness |
| *PgPRX17* | 91 | 98 | AuxRR-core | cis-acting regulatory element involved in auxin responsiveness |
| *PgPRX17* | 739 | 745 | G-Box | cis-acting regulatory element involved in light responsiveness |
| *PgPRX17* | 791 | 797 | G-Box | cis-acting regulatory element involved in light responsiveness |
| *PgPRX17* | 218 | 224 | CAT-box | cis-acting regulatory element related to meristem expression |
| *PgPRX17* | 935 | 941 | CAT-box | cis-acting regulatory element related to meristem expression |
| *PgPRX17* | 584 | 591 | GCN4_motif | cis-regulatory element involved in endosperm expression |
| *PgPRX17* | 111 | 116 | CAAT-box | common cis-acting element in promoter and enhancer regions |
| *PgPRX17* | 142 | 147 | CAAT-box | common cis-acting element in promoter and enhancer regions |
| *PgPRX17* | 197 | 202 | CAAT-box | common cis-acting element in promoter and enhancer regions |
| *PgPRX17* | 513 | 518 | CAAT-box | common cis-acting element in promoter and enhancer regions |
| *PgPRX17* | 551 | 556 | CAAT-box | common cis-acting element in promoter and enhancer regions |
| *PgPRX17* | 589 | 594 | CAAT-box | common cis-acting element in promoter and enhancer regions |
| *PgPRX17* | 631 | 636 | CAAT-box | common cis-acting element in promoter and enhancer regions |
| *PgPRX17* | 701 | 706 | CAAT-box | common cis-acting element in promoter and enhancer regions |
| *PgPRX17* | 785 | 790 | CAAT-box | common cis-acting element in promoter and enhancer regions |
| *PgPRX17* | 865 | 870 | CAAT-box | common cis-acting element in promoter and enhancer regions |
| *PgPRX17* | 168 | 176 | TCT-motif | part of a light responsive element |
| *PgPRX17* | 860 | 866 | TCT-motif | part of a light responsive element |
| *PgPRX18* | 436 | 442 | ABRE | cis-acting element involved in the abscisic acid responsiveness |
| *PgPRX18* | 437 | 442 | ABRE | cis-acting element involved in the abscisic acid responsiveness |
| *PgPRX18* | 719 | 725 | ARE | cis-acting regulatory element essential for the anaerobic induction |
| *PgPRX18* | 436 | 442 | G-Box | cis-acting regulatory element involved in light responsiveness |
| *PgPRX18* | 436 | 442 | G-Box | cis-acting regulatory element involved in light responsiveness |
| *PgPRX18* | 3 | 8 | CAAT-box | common cis-acting element in promoter and enhancer regions |
| *PgPRX18* | 32 | 37 | CAAT-box | common cis-acting element in promoter and enhancer regions |
| *PgPRX18* | 134 | 139 | CAAT-box | common cis-acting element in promoter and enhancer regions |
| *PgPRX18* | 394 | 399 | CAAT-box | common cis-acting element in promoter and enhancer regions |
| *PgPRX18* | 455 | 460 | CAAT-box | common cis-acting element in promoter and enhancer regions |
| *PgPRX18* | 538 | 543 | CAAT-box | common cis-acting element in promoter and enhancer regions |
| *PgPRX18* | 640 | 645 | CAAT-box | common cis-acting element in promoter and enhancer regions |
| *PgPRX18* | 746 | 751 | CAAT-box | common cis-acting element in promoter and enhancer regions |
| *PgPRX18* | 929 | 934 | CAAT-box | common cis-acting element in promoter and enhancer regions |
| *PgPRX18* | 970 | 975 | CAAT-box | common cis-acting element in promoter and enhancer regions |
| *PgPRX18* | 298 | 304 | GC-motif | enhancer-like element involved in anoxic specific inducibility |
| *PgPRX18* | 34 | 43 | Box 4 | part of a conserved DNA module involved in light responsiveness |
| *PgPRX18* | 11 | 17 | Box 4 | part of a conserved DNA module involved in light responsiveness |
| *PgPRX18* | 418 | 427 | TCT-motif | part of a light responsive element |
| *PgPRX18* | 697 | 704 | TCT-motif | part of a light responsive element |
| *PgPRX18* | 630 | 636 | TCT-motif | part of a light responsive element |
| *PgPRX19* | 171 | 176 | ABRE | cis-acting element involved in the abscisic acid responsiveness |
| *PgPRX19* | 245 | 250 | ABRE | cis-acting element involved in the abscisic acid responsiveness |
| *PgPRX19* | 844 | 853 | ABRE | cis-acting element involved in the abscisic acid responsiveness |
| *PgPRX19* | 207 | 213 | ARE | cis-acting regulatory element essential for the anaerobic induction |
| *PgPRX19* | 171 | 177 | G-Box | cis-acting regulatory element involved in light responsiveness |
| *PgPRX19* | 244 | 250 | G-Box | cis-acting regulatory element involved in light responsiveness |
| *PgPRX19* | 25 | 30 | CAAT-box | common cis-acting element in promoter and enhancer regions |
| *PgPRX19* | 121 | 126 | CAAT-box | common cis-acting element in promoter and enhancer regions |
| *PgPRX19* | 277 | 282 | CAAT-box | common cis-acting element in promoter and enhancer regions |
| *PgPRX19* | 307 | 312 | CAAT-box | common cis-acting element in promoter and enhancer regions |
| *PgPRX19* | 325 | 330 | CAAT-box | common cis-acting element in promoter and enhancer regions |
| *PgPRX19* | 364 | 369 | CAAT-box | common cis-acting element in promoter and enhancer regions |
| *PgPRX19* | 630 | 635 | CAAT-box | common cis-acting element in promoter and enhancer regions |
| *PgPRX19* | 969 | 974 | CAAT-box | common cis-acting element in promoter and enhancer regions |
| *PgPRX19* | 234 | 242 | Box 4 | part of a conserved DNA module involved in light responsiveness |
| *PgPRX19* | 350 | 359 | Box 4 | part of a conserved DNA module involved in light responsiveness |
| *PgPRX20* | 248 | 257 | ACE | cis-acting element involved in light responsiveness |
| *PgPRX20* | 187 | 192 | CAAT-box | common cis-acting element in promoter and enhancer regions |
| *PgPRX20* | 235 | 240 | CAAT-box | common cis-acting element in promoter and enhancer regions |
| *PgPRX20* | 544 | 549 | CAAT-box | common cis-acting element in promoter and enhancer regions |
| *PgPRX20* | 640 | 645 | CAAT-box | common cis-acting element in promoter and enhancer regions |
| *PgPRX20* | 652 | 657 | CAAT-box | common cis-acting element in promoter and enhancer regions |
| *PgPRX20* | 885 | 890 | CAAT-box | common cis-acting element in promoter and enhancer regions |
| *PgPRX20* | 661 | 667 | GC-motif | enhancer-like element involved in anoxic specific inducibility |
| *PgPRX21* | 472 | 481 | TCA-element | cis-acting element involved in salicylic acid responsiveness |
| *PgPRX21* | 57 | 63 | ABRE | cis-acting element involved in the abscisic acid responsiveness |
| *PgPRX21* | 58 | 63 | ABRE | cis-acting element involved in the abscisic acid responsiveness |
| *PgPRX21* | 875 | 880 | ABRE | cis-acting element involved in the abscisic acid responsiveness |
| *PgPRX21* | 502 | 508 | ARE | cis-acting regulatory element essential for the anaerobic induction |
| *PgPRX21* | 57 | 63 | G-Box | cis-acting regulatory element involved in light responsiveness |
| *PgPRX21* | 873 | 883 | G-Box | cis-acting regulatory element involved in light responsiveness |
| *PgPRX21* | 874 | 880 | G-Box | cis-acting regulatory element involved in light responsiveness |
| *PgPRX21* | 57 | 63 | G-Box | cis-acting regulatory element involved in light responsiveness |
| *PgPRX21* | 754 | 759 | TGACG-motif | cis-acting regulatory element involved in the MeJA-responsiveness |
| *PgPRX21* | 754 | 759 | TGACG-motif | cis-acting regulatory element involved in the MeJA-responsiveness |
| *PgPRX21* | 399 | 405 | CAT-box | cis-acting regulatory element related to meristem expression |
| *PgPRX21* | 490 | 497 | GCN4_motif | cis-regulatory element involved in endosperm expression |
| *PgPRX21* | 28 | 33 | CAAT-box | common cis-acting element in promoter and enhancer regions |
| *PgPRX21* | 68 | 73 | CAAT-box | common cis-acting element in promoter and enhancer regions |
| *PgPRX21* | 180 | 185 | CAAT-box | common cis-acting element in promoter and enhancer regions |
| *PgPRX21* | 185 | 190 | CAAT-box | common cis-acting element in promoter and enhancer regions |
| *PgPRX21* | 696 | 701 | CAAT-box | common cis-acting element in promoter and enhancer regions |
| *PgPRX21* | 808 | 813 | CAAT-box | common cis-acting element in promoter and enhancer regions |
| *PgPRX21* | 822 | 827 | CAAT-box | common cis-acting element in promoter and enhancer regions |
| *PgPRX21* | 834 | 841 | P-box | gibberellin-responsive element |
| *PgPRX21* | 120 | 126 | GT1-motif | light responsive element |
| *PgPRX21* | 71 | 77 | Box 4 | part of a conserved DNA module involved in light responsiveness |
| *PgPRX21* | 957 | 965 | TCT-motif | part of a light responsive element |
| *PgPRX21* | 872 | 881 | TCT-motif | part of a light responsive element |
| *PgPRX21* | 696 | 705.5 | TCT-motif | part of a light responsive element |
| *PgPRX22* | 44 | 50 | TGA-element | auxin-responsive element |
| *PgPRX22* | 86 | 91 | ABRE | cis-acting element involved in the abscisic acid responsiveness |
| *PgPRX22* | 143 | 149 | ABRE | cis-acting element involved in the abscisic acid responsiveness |
| *PgPRX22* | 144 | 149 | ABRE | cis-acting element involved in the abscisic acid responsiveness |
| *PgPRX22* | 314 | 319 | ABRE | cis-acting element involved in the abscisic acid responsiveness |
| *PgPRX22* | 942 | 947 | ABRE | cis-acting element involved in the abscisic acid responsiveness |
| *PgPRX22* | 746 | 752 | ARE | cis-acting regulatory element essential for the anaerobic induction |
| *PgPRX22* | 143 | 149 | G-Box | cis-acting regulatory element involved in light responsiveness |
| *PgPRX22* | 313 | 319 | G-Box | cis-acting regulatory element involved in light responsiveness |
| *PgPRX22* | 86 | 92 | G-Box | cis-acting regulatory element involved in light responsiveness |
| *PgPRX22* | 143 | 149 | G-Box | cis-acting regulatory element involved in light responsiveness |
| *PgPRX22* | 311 | 319 | G-Box | cis-acting regulatory element involved in light responsiveness |
| *PgPRX22* | 705 | 711 | G-Box | cis-acting regulatory element involved in light responsiveness |
| *PgPRX22* | 942 | 948 | G-Box | cis-acting regulatory element involved in light responsiveness |
| *PgPRX22* | 40 | 45 | TGACG-motif | cis-acting regulatory element involved in the MeJA-responsiveness |
| *PgPRX22* | 40 | 45 | TGACG-motif | cis-acting regulatory element involved in the MeJA-responsiveness |
| *PgPRX22* | 37 | 46 | O2-site | cis-acting regulatory element involved in zein metabolism regulation |
| *PgPRX22* | 508 | 513 | CAAT-box | common cis-acting element in promoter and enhancer regions |
| *PgPRX22* | 593 | 598 | CAAT-box | common cis-acting element in promoter and enhancer regions |
| *PgPRX22* | 684 | 689 | CAAT-box | common cis-acting element in promoter and enhancer regions |
| *PgPRX22* | 700 | 705 | CAAT-box | common cis-acting element in promoter and enhancer regions |
| *PgPRX22* | 740 | 745 | CAAT-box | common cis-acting element in promoter and enhancer regions |
| *PgPRX22* | 907 | 912 | CAAT-box | common cis-acting element in promoter and enhancer regions |
| *PgPRX22* | 980 | 985 | CAAT-box | common cis-acting element in promoter and enhancer regions |
| *PgPRX22* | 31 | 37 | GC-motif | enhancer-like element involved in anoxic specific inducibility |
| *PgPRX22* | 419 | 425 | GT1-motif | light responsive element |
| *PgPRX22* | 456 | 463 | GT1-motif | light responsive element |
| *PgPRX22* | 457 | 463 | GT1-motif | light responsive element |
| *PgPRX22* | 609 | 615 | GT1-motif | light responsive element |
| *PgPRX22* | 781 | 787 | GT1-motif | light responsive element |
| *PgPRX22* | 24 | 30 | TCT-motif | part of a light responsive element |
| *PgPRX22* | 299 | 308 | TCT-motif | part of a light responsive element |
| *PgPRX22* | 959 | 966 | TCT-motif | part of a light responsive element |
| *PgPRX22* | 297 | 306 | TCT-motif | part of a light responsive element |
| *PgPRX22* | 37 | 45 | AuxRE | part of an auxin-responsive element |
| *PgPRX23* | 979 | 985 | A-box | cis-acting regulatory element |
| *PgPRX23* | 328 | 334 | ARE | cis-acting regulatory element essential for the anaerobic induction |
| *PgPRX23* | 278 | 287 | circadian | cis-acting regulatory element involved in circadian control |
| *PgPRX23* | 388 | 393 | TGACG-motif | cis-acting regulatory element involved in the MeJA-responsiveness |
| *PgPRX23* | 388 | 393 | TGACG-motif | cis-acting regulatory element involved in the MeJA-responsiveness |
| *PgPRX23* | 832 | 841 | O2-site | cis-acting regulatory element involved in zein metabolism regulation |
| *PgPRX23* | 913 | 921 | O2-site | cis-acting regulatory element involved in zein metabolism regulation |
| *PgPRX23* | 28 | 33 | CAAT-box | common cis-acting element in promoter and enhancer regions |
| *PgPRX23* | 393 | 398 | CAAT-box | common cis-acting element in promoter and enhancer regions |
| *PgPRX23* | 408 | 413 | CAAT-box | common cis-acting element in promoter and enhancer regions |
| *PgPRX23* | 411 | 416 | CAAT-box | common cis-acting element in promoter and enhancer regions |
| *PgPRX23* | 493 | 498 | CAAT-box | common cis-acting element in promoter and enhancer regions |
| *PgPRX23* | 804 | 809 | CAAT-box | common cis-acting element in promoter and enhancer regions |
| *PgPRX23* | 602 | 609 | P-box | gibberellin-responsive element |
| *PgPRX23* | 965 | 972 | P-box | gibberellin-responsive element |
| *PgPRX23* | 425 | 431 | Box 4 | part of a conserved DNA module involved in light responsiveness |
| *PgPRX24* | 523 | 529 | TGA-element | auxin-responsive element |
| *PgPRX24* | 759 | 765 | TGA-element | auxin-responsive element |
| *PgPRX24* | 519 | 525 | LTR | cis-acting element involved in low-temperature responsiveness |
| *PgPRX24* | 489 | 498 | TCA-element | cis-acting element involved in salicylic acid responsiveness |
| *PgPRX24* | 514 | 521 | AuxRR-core | cis-acting regulatory element involved in auxin responsiveness |
| *PgPRX24* | 314 | 319 | TGACG-motif | cis-acting regulatory element involved in the MeJA-responsiveness |
| *PgPRX24* | 370 | 375 | TGACG-motif | cis-acting regulatory element involved in the MeJA-responsiveness |
| *PgPRX24* | 314 | 319 | TGACG-motif | cis-acting regulatory element involved in the MeJA-responsiveness |
| *PgPRX24* | 370 | 375 | TGACG-motif | cis-acting regulatory element involved in the MeJA-responsiveness |
| *PgPRX24* | 5 | 10 | CAAT-box | common cis-acting element in promoter and enhancer regions |
| *PgPRX24* | 59 | 64 | CAAT-box | common cis-acting element in promoter and enhancer regions |
| *PgPRX24* | 123 | 128 | CAAT-box | common cis-acting element in promoter and enhancer regions |
| *PgPRX24* | 165 | 170 | CAAT-box | common cis-acting element in promoter and enhancer regions |
| *PgPRX24* | 235 | 240 | CAAT-box | common cis-acting element in promoter and enhancer regions |
| *PgPRX24* | 299 | 304 | CAAT-box | common cis-acting element in promoter and enhancer regions |
| *PgPRX24* | 305 | 310 | CAAT-box | common cis-acting element in promoter and enhancer regions |
| *PgPRX24* | 461 | 466 | CAAT-box | common cis-acting element in promoter and enhancer regions |
| *PgPRX24* | 621 | 626 | CAAT-box | common cis-acting element in promoter and enhancer regions |
| *PgPRX24* | 741 | 746 | CAAT-box | common cis-acting element in promoter and enhancer regions |
| *PgPRX24* | 747 | 758 | CAAT-box | common cis-acting element in promoter and enhancer regions |
| *PgPRX24* | 826 | 831 | CAAT-box | common cis-acting element in promoter and enhancer regions |
| *PgPRX24* | 829 | 834 | CAAT-box | common cis-acting element in promoter and enhancer regions |
| *PgPRX24* | 856 | 861 | CAAT-box | common cis-acting element in promoter and enhancer regions |
| *PgPRX24* | 430 | 436 | GC-motif | enhancer-like element involved in anoxic specific inducibility |
| *PgPRX24* | 40 | 49 | Box 4 | part of a conserved DNA module involved in light responsiveness |
| *PgPRX24* | 842 | 849 | TCT-motif | part of a light responsive element |
| *PgPRX25* | 170 | 179 | TC-rich repeats | cis-acting element involved in defense and stress responsiveness |
| *PgPRX25* | 916 | 922 | LTR | cis-acting element involved in low-temperature responsiveness |
| *PgPRX25* | 592 | 601 | TCA-element | cis-acting element involved in salicylic acid responsiveness |
| *PgPRX25* | 376 | 382 | G-Box | cis-acting regulatory element involved in light responsiveness |
| *PgPRX25* | 357 | 362 | TGACG-motif | cis-acting regulatory element involved in the MeJA-responsiveness |
| *PgPRX25* | 357 | 362 | TGACG-motif | cis-acting regulatory element involved in the MeJA-responsiveness |
| *PgPRX25* | 22 | 27 | CAAT-box | common cis-acting element in promoter and enhancer regions |
| *PgPRX25* | 327 | 332 | CAAT-box | common cis-acting element in promoter and enhancer regions |
| *PgPRX25* | 345 | 350 | CAAT-box | common cis-acting element in promoter and enhancer regions |
| *PgPRX25* | 404 | 409 | CAAT-box | common cis-acting element in promoter and enhancer regions |
| *PgPRX25* | 626 | 631 | CAAT-box | common cis-acting element in promoter and enhancer regions |
| *PgPRX25* | 705 | 710 | CAAT-box | common cis-acting element in promoter and enhancer regions |
| *PgPRX25* | 552 | 559 | P-box | gibberellin-responsive element |
| *PgPRX25* | 204 | 210 | Box 4 | part of a conserved DNA module involved in light responsiveness |
| *PgPRX25* | 170 | 176 | TCT-motif | part of a light responsive element |
| *PgPRX25* | 291 | 297 | TCT-motif | part of a light responsive element |
| *PgPRX25* | 207 | 217 | TCT-motif | part of a light responsive element |
| *PgPRX25* | 209 | 216 | TCT-motif | part of a light responsive element |
| *PgPRX26* | 156 | 162 | LTR | cis-acting element involved in low-temperature responsiveness |
| *PgPRX26* | 667 | 675 | ABRE | cis-acting element involved in the abscisic acid responsiveness |
| *PgPRX26* | 668 | 673 | ABRE | cis-acting element involved in the abscisic acid responsiveness |
| *PgPRX26* | 743 | 748 | ABRE | cis-acting element involved in the abscisic acid responsiveness |
| *PgPRX26* | 201 | 207 | ARE | cis-acting regulatory element essential for the anaerobic induction |
| *PgPRX26* | 216 | 222 | ARE | cis-acting regulatory element essential for the anaerobic induction |
| *PgPRX26* | 621 | 627 | ARE | cis-acting regulatory element essential for the anaerobic induction |
| *PgPRX26* | 513 | 523 | circadian | cis-acting regulatory element involved in circadian control |
| *PgPRX26* | 667 | 673 | G-Box | cis-acting regulatory element involved in light responsiveness |
| *PgPRX26* | 743 | 749 | G-Box | cis-acting regulatory element involved in light responsiveness |
| *PgPRX26* | 225 | 234 | O2-site | cis-acting regulatory element involved in zein metabolism regulation |
| *PgPRX26* | 654 | 660 | CAT-box | cis-acting regulatory element related to meristem expression |
| *PgPRX26* | 915 | 921 | CAT-box | cis-acting regulatory element related to meristem expression |
| *PgPRX26* | 137 | 142 | CAAT-box | common cis-acting element in promoter and enhancer regions |
| *PgPRX26* | 301 | 306 | CAAT-box | common cis-acting element in promoter and enhancer regions |
| *PgPRX26* | 624 | 629 | CAAT-box | common cis-acting element in promoter and enhancer regions |
| *PgPRX26* | 725 | 730 | CAAT-box | common cis-acting element in promoter and enhancer regions |
| *PgPRX26* | 688 | 694 | GC-motif | enhancer-like element involved in anoxic specific inducibility |
| *PgPRX26* | 649 | 659 | TCT-motif | part of a light responsive element |
| *PgPRX26* | 756 | 764 | TCT-motif | part of a light responsive element |
| *PgPRX27* | 181 | 187 | TGA-element | auxin-responsive element |
| *PgPRX27* | 169 | 176 | ABRE | cis-acting element involved in the abscisic acid responsiveness |
| *PgPRX27* | 755 | 764 | ABRE | cis-acting element involved in the abscisic acid responsiveness |
| *PgPRX27* | 773 | 779 | ARE | cis-acting regulatory element essential for the anaerobic induction |
| *PgPRX27* | 763 | 771 | RY-element | cis-acting regulatory element involved in seed-specific regulation |
| *PgPRX27* | 767 | 775 | RY-element | cis-acting regulatory element involved in seed-specific regulation |
| *PgPRX27* | 823 | 828 | TGACG-motif | cis-acting regulatory element involved in the MeJA-responsiveness |
| *PgPRX27* | 823 | 828 | TGACG-motif | cis-acting regulatory element involved in the MeJA-responsiveness |
| *PgPRX27* | 140 | 145 | CAAT-box | common cis-acting element in promoter and enhancer regions |
| *PgPRX27* | 505 | 510 | CAAT-box | common cis-acting element in promoter and enhancer regions |
| *PgPRX27* | 510 | 515 | CAAT-box | common cis-acting element in promoter and enhancer regions |
| *PgPRX27* | 515 | 520 | CAAT-box | common cis-acting element in promoter and enhancer regions |
| *PgPRX27* | 680 | 685 | CAAT-box | common cis-acting element in promoter and enhancer regions |
| *PgPRX27* | 746 | 751 | CAAT-box | common cis-acting element in promoter and enhancer regions |
| *PgPRX27* | 648 | 654 | Box 4 | part of a conserved DNA module involved in light responsiveness |
| *PgPRX27* | 78 | 85 | TCT-motif | part of a light responsive element |
| *PgPRX27* | 165 | 171 | TCT-motif | part of a light responsive element |
| *PgPRX27* | 173 | 179 | TCT-motif | part of a light responsive element |
| *PgPRX27* | 187 | 198 | AuxRE | part of an auxin-responsive element |
| *PgPRX28* | 264 | 274 | ACE | cis-acting element involved in light responsiveness |
| *PgPRX28* | 274 | 283 | TCA-element | cis-acting element involved in salicylic acid responsiveness |
| *PgPRX28* | 326 | 335 | TCA-element | cis-acting element involved in salicylic acid responsiveness |
| *PgPRX28* | 893 | 898 | ABRE | cis-acting element involved in the abscisic acid responsiveness |
| *PgPRX28* | 281 | 287 | ARE | cis-acting regulatory element essential for the anaerobic induction |
| *PgPRX28* | 920 | 926 | ARE | cis-acting regulatory element essential for the anaerobic induction |
| *PgPRX28* | 893 | 899 | G-Box | cis-acting regulatory element involved in light responsiveness |
| *PgPRX28* | 578 | 583 | TGACG-motif | cis-acting regulatory element involved in the MeJA-responsiveness |
| *PgPRX28* | 578 | 583 | TGACG-motif | cis-acting regulatory element involved in the MeJA-responsiveness |
| *PgPRX28* | 14 | 19 | CAAT-box | common cis-acting element in promoter and enhancer regions |
| *PgPRX28* | 38 | 43 | CAAT-box | common cis-acting element in promoter and enhancer regions |
| *PgPRX28* | 63 | 68 | CAAT-box | common cis-acting element in promoter and enhancer regions |
| *PgPRX28* | 112 | 117 | CAAT-box | common cis-acting element in promoter and enhancer regions |
| *PgPRX28* | 943 | 948 | CAAT-box | common cis-acting element in promoter and enhancer regions |
| *PgPRX28* | 168 | 174 | GC-motif | enhancer-like element involved in anoxic specific inducibility |
| *PgPRX28* | 822 | 828 | GC-motif | enhancer-like element involved in anoxic specific inducibility |
| *PgPRX28* | 561 | 567 | Box 4 | part of a conserved DNA module involved in light responsiveness |
| *PgPRX28* | 71 | 78 | TCT-motif | part of a light responsive element |
| *PgPRX29* | 109 | 115 | ABRE | cis-acting element involved in the abscisic acid responsiveness |
| *PgPRX29* | 110 | 115 | ABRE | cis-acting element involved in the abscisic acid responsiveness |
| *PgPRX29* | 373 | 379 | ARE | cis-acting regulatory element essential for the anaerobic induction |
| *PgPRX29* | 109 | 115 | G-Box | cis-acting regulatory element involved in light responsiveness |
| *PgPRX29* | 109 | 115 | G-Box | cis-acting regulatory element involved in light responsiveness |
| *PgPRX29* | 731 | 740 | G-Box | cis-acting regulatory element involved in light responsiveness |
| *PgPRX29* | 964 | 970 | G-Box | cis-acting regulatory element involved in light responsiveness |
| *PgPRX29* | 837 | 846 | O2-site | cis-acting regulatory element involved in zein metabolism regulation |
| *PgPRX29* | 131 | 136 | CAAT-box | common cis-acting element in promoter and enhancer regions |
| *PgPRX29* | 241 | 246 | CAAT-box | common cis-acting element in promoter and enhancer regions |
| *PgPRX29* | 284 | 289 | CAAT-box | common cis-acting element in promoter and enhancer regions |
| *PgPRX29* | 371 | 376 | CAAT-box | common cis-acting element in promoter and enhancer regions |
| *PgPRX29* | 414 | 419 | CAAT-box | common cis-acting element in promoter and enhancer regions |
| *PgPRX29* | 602 | 607 | CAAT-box | common cis-acting element in promoter and enhancer regions |
| *PgPRX29* | 880 | 885 | CAAT-box | common cis-acting element in promoter and enhancer regions |
| *PgPRX29* | 47 | 54 | P-box | gibberellin-responsive element |
| *PgPRX29* | 479 | 486 | P-box | gibberellin-responsive element |
| *PgPRX29* | 58 | 64 | Box 4 | part of a conserved DNA module involved in light responsiveness |
| *PgPRX29* | 239 | 248 | Box 4 | part of a conserved DNA module involved in light responsiveness |
| *PgPRX29* | 282 | 291 | Box 4 | part of a conserved DNA module involved in light responsiveness |
| *PgPRX29* | 676 | 682 | TCT-motif | part of a light responsive element |
| *PgPRX29* | 74 | 82 | AE-box | part of a module for light response |
| *PgPRX29* | 451 | 459 | AE-box | part of a module for light response |
| *PgPRX30* | 969 | 975 | TGA-element | auxin-responsive element |
| *PgPRX30* | 683 | 690 | TATC-box | cis-acting element involved in gibberellin-responsiveness |
| *PgPRX30* | 347 | 353 | LTR | cis-acting element involved in low-temperature responsiveness |
| *PgPRX30* | 779 | 785 | LTR | cis-acting element involved in low-temperature responsiveness |
| *PgPRX30* | 687 | 696 | TCA-element | cis-acting element involved in salicylic acid responsiveness |
| *PgPRX30* | 478 | 483 | ABRE | cis-acting element involved in the abscisic acid responsiveness |
| *PgPRX30* | 652 | 657 | ABRE | cis-acting element involved in the abscisic acid responsiveness |
| *PgPRX30* | 478 | 484 | G-Box | cis-acting regulatory element involved in light responsiveness |
| *PgPRX30* | 273 | 279 | G-Box | cis-acting regulatory element involved in light responsiveness |
| *PgPRX30* | 478 | 486 | G-Box | cis-acting regulatory element involved in light responsiveness |
| *PgPRX30* | 652 | 658 | G-Box | cis-acting regulatory element involved in light responsiveness |
| *PgPRX30* | 717 | 723 | G-Box | cis-acting regulatory element involved in light responsiveness |
| *PgPRX30* | 654 | 659 | TGACG-motif | cis-acting regulatory element involved in the MeJA-responsiveness |
| *PgPRX30* | 654 | 659 | TGACG-motif | cis-acting regulatory element involved in the MeJA-responsiveness |
| *PgPRX30* | 651 | 660 | O2-site | cis-acting regulatory element involved in zein metabolism regulation |
| *PgPRX30* | 23 | 31 | CAAT-box | common cis-acting element in promoter and enhancer regions |
| *PgPRX30* | 24 | 29 | CAAT-box | common cis-acting element in promoter and enhancer regions |
| *PgPRX30* | 59 | 64 | CAAT-box | common cis-acting element in promoter and enhancer regions |
| *PgPRX30* | 83 | 88 | CAAT-box | common cis-acting element in promoter and enhancer regions |
| *PgPRX30* | 86 | 91 | CAAT-box | common cis-acting element in promoter and enhancer regions |
| *PgPRX30* | 186 | 191 | CAAT-box | common cis-acting element in promoter and enhancer regions |
| *PgPRX30* | 209 | 214 | CAAT-box | common cis-acting element in promoter and enhancer regions |
| *PgPRX30* | 217 | 222 | CAAT-box | common cis-acting element in promoter and enhancer regions |
| *PgPRX30* | 446 | 451 | CAAT-box | common cis-acting element in promoter and enhancer regions |
| *PgPRX30* | 609 | 614 | CAAT-box | common cis-acting element in promoter and enhancer regions |
| *PgPRX30* | 657 | 662 | CAAT-box | common cis-acting element in promoter and enhancer regions |
| *PgPRX30* | 672 | 677 | CAAT-box | common cis-acting element in promoter and enhancer regions |
| *PgPRX30* | 198 | 207 | GT1-motif | light responsive element |
| *PgPRX30* | 51 | 57 | GT1-motif | light responsive element |
| *PgPRX30* | 394 | 403 | Box 4 | part of a conserved DNA module involved in light responsiveness |
| *PgPRX31* | 79 | 85 | ARE | cis-acting regulatory element essential for the anaerobic induction |
| *PgPRX31* | 470 | 475 | TGACG-motif | cis-acting regulatory element involved in the MeJA-responsiveness |
| *PgPRX31* | 470 | 475 | TGACG-motif | cis-acting regulatory element involved in the MeJA-responsiveness |
| *PgPRX31* | 443 | 449 | CAT-box | cis-acting regulatory element related to meristem expression |
| *PgPRX31* | 280 | 285 | CAAT-box | common cis-acting element in promoter and enhancer regions |
| *PgPRX31* | 293 | 298 | CAAT-box | common cis-acting element in promoter and enhancer regions |
| *PgPRX31* | 615 | 620 | CAAT-box | common cis-acting element in promoter and enhancer regions |
| *PgPRX31* | 485 | 491 | GT1-motif | light responsive element |
| *PgPRX31* | 658 | 664 | Box 4 | part of a conserved DNA module involved in light responsiveness |
| *PgPRX31* | 663 | 669 | TCT-motif | part of a light responsive element |
| *PgPRX31* | 680 | 686 | TCT-motif | part of a light responsive element |
| *PgPRX31* | 719 | 726 | TCT-motif | part of a light responsive element |
| *PgPRX31* | 910 | 918 | TCT-motif | part of a light responsive element |
| *PgPRX31* | 95 | 104 | TCT-motif | part of a light responsive element |
| *PgPRX32* | 740 | 746 | LTR | cis-acting element involved in low-temperature responsiveness |
| *PgPRX32* | 772 | 781 | ABRE | cis-acting element involved in the abscisic acid responsiveness |
| *PgPRX32* | 774 | 780 | ABRE | cis-acting element involved in the abscisic acid responsiveness |
| *PgPRX32* | 775 | 780 | ABRE | cis-acting element involved in the abscisic acid responsiveness |
| *PgPRX32* | 880 | 886 | ARE | cis-acting regulatory element essential for the anaerobic induction |
| *PgPRX32* | 912 | 918 | ARE | cis-acting regulatory element essential for the anaerobic induction |
| *PgPRX32* | 139 | 146 | AuxRR-core | cis-acting regulatory element involved in auxin responsiveness |
| *PgPRX32* | 114 | 123 | circadian | cis-acting regulatory element involved in circadian control |
| *PgPRX32* | 118 | 127 | circadian | cis-acting regulatory element involved in circadian control |
| *PgPRX32* | 774 | 780 | G-Box | cis-acting regulatory element involved in light responsiveness |
| *PgPRX32* | 774 | 780 | G-Box | cis-acting regulatory element involved in light responsiveness |
| *PgPRX32* | 874 | 882 | RY-element | cis-acting regulatory element involved in seed-specific regulation |
| *PgPRX32* | 612 | 617 | TGACG-motif | cis-acting regulatory element involved in the MeJA-responsiveness |
| *PgPRX32* | 612 | 617 | TGACG-motif | cis-acting regulatory element involved in the MeJA-responsiveness |
| *PgPRX32* | 313 | 318 | CAAT-box | common cis-acting element in promoter and enhancer regions |
| *PgPRX32* | 369 | 374 | CAAT-box | common cis-acting element in promoter and enhancer regions |
| *PgPRX32* | 429 | 434 | CAAT-box | common cis-acting element in promoter and enhancer regions |
| *PgPRX32* | 452 | 457 | CAAT-box | common cis-acting element in promoter and enhancer regions |
| *PgPRX32* | 561 | 566 | CAAT-box | common cis-acting element in promoter and enhancer regions |
| *PgPRX32* | 599 | 604 | CAAT-box | common cis-acting element in promoter and enhancer regions |
| *PgPRX32* | 689 | 694 | CAAT-box | common cis-acting element in promoter and enhancer regions |
| *PgPRX32* | 984 | 989 | CAAT-box | common cis-acting element in promoter and enhancer regions |
| *PgPRX32* | 929 | 935 | GT1-motif | light responsive element |
| *PgPRX32* | 36 | 43 | TCT-motif | part of a light responsive element |
| *PgPRX33* | 82 | 91 | TC-rich repeats | cis-acting element involved in defense and stress responsiveness |
| *PgPRX33* | 726 | 735 | ACE | cis-acting element involved in light responsiveness |
| *PgPRX33* | 875 | 881 | LTR | cis-acting element involved in low-temperature responsiveness |
| *PgPRX33* | 57 | 62 | ABRE | cis-acting element involved in the abscisic acid responsiveness |
| *PgPRX33* | 238 | 243 | ABRE | cis-acting element involved in the abscisic acid responsiveness |
| *PgPRX33* | 377 | 383 | ARE | cis-acting regulatory element essential for the anaerobic induction |
| *PgPRX33* | 608 | 614 | ARE | cis-acting regulatory element essential for the anaerobic induction |
| *PgPRX33* | 476 | 485 | circadian | cis-acting regulatory element involved in circadian control |
| *PgPRX33* | 56 | 62 | G-Box | cis-acting regulatory element involved in light responsiveness |
| *PgPRX33* | 238 | 244 | G-Box | cis-acting regulatory element involved in light responsiveness |
| *PgPRX33* | 987 | 992 | TGACG-motif | cis-acting regulatory element involved in the MeJA-responsiveness |
| *PgPRX33* | 987 | 992 | TGACG-motif | cis-acting regulatory element involved in the MeJA-responsiveness |
| *PgPRX33* | 320 | 329 | O2-site | cis-acting regulatory element involved in zein metabolism regulation |
| *PgPRX33* | 19 | 24 | CAAT-box | common cis-acting element in promoter and enhancer regions |
| *PgPRX33* | 287 | 292 | CAAT-box | common cis-acting element in promoter and enhancer regions |
| *PgPRX33* | 307 | 312 | CAAT-box | common cis-acting element in promoter and enhancer regions |
| *PgPRX33* | 370 | 375 | CAAT-box | common cis-acting element in promoter and enhancer regions |
| *PgPRX33* | 633 | 638 | CAAT-box | common cis-acting element in promoter and enhancer regions |
| *PgPRX33* | 652 | 657 | CAAT-box | common cis-acting element in promoter and enhancer regions |
| *PgPRX33* | 747 | 752 | CAAT-box | common cis-acting element in promoter and enhancer regions |
| *PgPRX33* | 797 | 802 | CAAT-box | common cis-acting element in promoter and enhancer regions |
| *PgPRX33* | 956 | 961 | CAAT-box | common cis-acting element in promoter and enhancer regions |
| *PgPRX33* | 115 | 121 | GC-motif | enhancer-like element involved in anoxic specific inducibility |
| *PgPRX34* | 314 | 323 | TC-rich repeats | cis-acting element involved in defense and stress responsiveness |
| *PgPRX34* | 771 | 778 | TATC-box | cis-acting element involved in gibberellin-responsiveness |
| *PgPRX34* | 555 | 561 | LTR | cis-acting element involved in low-temperature responsiveness |
| *PgPRX34* | 456 | 462 | ARE | cis-acting regulatory element essential for the anaerobic induction |
| *PgPRX34* | 552 | 558 | ARE | cis-acting regulatory element essential for the anaerobic induction |
| *PgPRX34* | 149 | 155 | G-Box | cis-acting regulatory element involved in light responsiveness |
| *PgPRX34* | 838 | 843 | TGACG-motif | cis-acting regulatory element involved in the MeJA-responsiveness |
| *PgPRX34* | 838 | 843 | TGACG-motif | cis-acting regulatory element involved in the MeJA-responsiveness |
| *PgPRX34* | 48 | 53 | CAAT-box | common cis-acting element in promoter and enhancer regions |
| *PgPRX34* | 127 | 132 | CAAT-box | common cis-acting element in promoter and enhancer regions |
| *PgPRX34* | 155 | 160 | CAAT-box | common cis-acting element in promoter and enhancer regions |
| *PgPRX34* | 201 | 206 | CAAT-box | common cis-acting element in promoter and enhancer regions |
| *PgPRX34* | 223 | 228 | CAAT-box | common cis-acting element in promoter and enhancer regions |
| *PgPRX34* | 284 | 289 | CAAT-box | common cis-acting element in promoter and enhancer regions |
| *PgPRX34* | 353 | 358 | CAAT-box | common cis-acting element in promoter and enhancer regions |
| *PgPRX34* | 378 | 383 | CAAT-box | common cis-acting element in promoter and enhancer regions |
| *PgPRX34* | 451 | 456 | CAAT-box | common cis-acting element in promoter and enhancer regions |
| *PgPRX34* | 682 | 687 | CAAT-box | common cis-acting element in promoter and enhancer regions |
| *PgPRX34* | 729 | 734 | CAAT-box | common cis-acting element in promoter and enhancer regions |
| *PgPRX34* | 769 | 774 | CAAT-box | common cis-acting element in promoter and enhancer regions |
| *PgPRX34* | 908 | 913 | CAAT-box | common cis-acting element in promoter and enhancer regions |
| *PgPRX34* | 563 | 569 | GC-motif | enhancer-like element involved in anoxic specific inducibility |
| *PgPRX34* | 509 | 517 | TCT-motif | part of a light responsive element |
| *PgPRX34* | 318 | 324 | TCT-motif | part of a light responsive element |
| *PgPRX34* | 825 | 832 | TCT-motif | part of a light responsive element |
| *PgPRX34* | 886 | 893 | TCT-motif | part of a light responsive element |
| *PgPRX35* | 573 | 579 | TGA-element | auxin-responsive element |
| *PgPRX35* | 81 | 90 | TC-rich repeats | cis-acting element involved in defense and stress responsiveness |
| *PgPRX35* | 201 | 206 | ABRE | cis-acting element involved in the abscisic acid responsiveness |
| *PgPRX35* | 211 | 216 | ABRE | cis-acting element involved in the abscisic acid responsiveness |
| *PgPRX35* | 61 | 67 | ARE | cis-acting regulatory element essential for the anaerobic induction |
| *PgPRX35* | 198 | 207 | G-Box | cis-acting regulatory element involved in light responsiveness |
| *PgPRX35* | 200 | 206 | G-Box | cis-acting regulatory element involved in light responsiveness |
| *PgPRX35* | 211 | 217 | G-Box | cis-acting regulatory element involved in light responsiveness |
| *PgPRX35* | 142 | 147 | TGACG-motif | cis-acting regulatory element involved in the MeJA-responsiveness |
| *PgPRX35* | 142 | 147 | TGACG-motif | cis-acting regulatory element involved in the MeJA-responsiveness |
| *PgPRX35* | 36 | 41 | CAAT-box | common cis-acting element in promoter and enhancer regions |
| *PgPRX35* | 69 | 74 | CAAT-box | common cis-acting element in promoter and enhancer regions |
| *PgPRX35* | 274 | 279 | CAAT-box | common cis-acting element in promoter and enhancer regions |
| *PgPRX35* | 407 | 412 | CAAT-box | common cis-acting element in promoter and enhancer regions |
| *PgPRX35* | 475 | 480 | CAAT-box | common cis-acting element in promoter and enhancer regions |
| *PgPRX35* | 729 | 734 | CAAT-box | common cis-acting element in promoter and enhancer regions |
| *PgPRX35* | 753 | 758 | CAAT-box | common cis-acting element in promoter and enhancer regions |
| *PgPRX35* | 919 | 924 | CAAT-box | common cis-acting element in promoter and enhancer regions |
| *PgPRX35* | 617 | 624 | P-box | gibberellin-responsive element |
| *PgPRX35* | 226 | 233 | TCT-motif | part of a light responsive element |
| *PgPRX35* | 899 | 906 | TCT-motif | part of a light responsive element |
| *PgPRX35* | 379 | 387 | AE-box | part of a module for light response |
| *PgPRX36* | 610 | 616 | LTR | cis-acting element involved in low-temperature responsiveness |
| *PgPRX36* | 797 | 807 | G-Box | cis-acting regulatory element involved in light responsiveness |
| *PgPRX36* | 740 | 745 | TGACG-motif | cis-acting regulatory element involved in the MeJA-responsiveness |
| *PgPRX36* | 740 | 745 | TGACG-motif | cis-acting regulatory element involved in the MeJA-responsiveness |
| *PgPRX36* | 549 | 554 | CAAT-box | common cis-acting element in promoter and enhancer regions |
| *PgPRX36* | 556 | 561 | CAAT-box | common cis-acting element in promoter and enhancer regions |
| *PgPRX36* | 684 | 689 | CAAT-box | common cis-acting element in promoter and enhancer regions |
| *PgPRX36* | 726 | 731 | CAAT-box | common cis-acting element in promoter and enhancer regions |
| *PgPRX36* | 732 | 737 | CAAT-box | common cis-acting element in promoter and enhancer regions |
| *PgPRX36* | 874 | 879 | CAAT-box | common cis-acting element in promoter and enhancer regions |
| *PgPRX36* | 692 | 701 | Box 4 | part of a conserved DNA module involved in light responsiveness |
| *PgPRX36* | 552 | 558 | Box 4 | part of a conserved DNA module involved in light responsiveness |
| *PgPRX37* | 819 | 825 | LTR | cis-acting element involved in low-temperature responsiveness |
| *PgPRX37* | 280 | 289 | ABRE | cis-acting element involved in the abscisic acid responsiveness |
| *PgPRX37* | 714 | 719 | ABRE | cis-acting element involved in the abscisic acid responsiveness |
| *PgPRX37* | 833 | 838 | ABRE | cis-acting element involved in the abscisic acid responsiveness |
| *PgPRX37* | 433 | 439 | ARE | cis-acting regulatory element essential for the anaerobic induction |
| *PgPRX37* | 575 | 581 | ARE | cis-acting regulatory element essential for the anaerobic induction |
| *PgPRX37* | 713 | 719 | G-Box | cis-acting regulatory element involved in light responsiveness |
| *PgPRX37* | 833 | 839 | G-Box | cis-acting regulatory element involved in light responsiveness |
| *PgPRX37* | 883 | 888 | TGACG-motif | cis-acting regulatory element involved in the MeJA-responsiveness |
| *PgPRX37* | 883 | 888 | TGACG-motif | cis-acting regulatory element involved in the MeJA-responsiveness |
| *PgPRX37* | 120 | 125 | CAAT-box | common cis-acting element in promoter and enhancer regions |
| *PgPRX37* | 271 | 276 | CAAT-box | common cis-acting element in promoter and enhancer regions |
| *PgPRX37* | 430 | 435 | CAAT-box | common cis-acting element in promoter and enhancer regions |
| *PgPRX37* | 802 | 809 | P-box | gibberellin-responsive element |
| *PgPRX37* | 445 | 451 | Box 4 | part of a conserved DNA module involved in light responsiveness |
| *PgPRX37* | 474 | 481 | TCT-motif | part of a light responsive element |
| *PgPRX37* | 848 | 858 | TCT-motif | part of a light responsive element |
| *PgPRX37* | 48 | 61 | AT1-motif | part of a light responsive module |
| *PgPRX37* | 763 | 771 | AE-box | part of a module for light response |
